# HOA2.0-ComPaRe: A next generation Harvard-Oxford Atlas comparative parcellation reasoning method for human and macaque individual brain parcellation and atlases of the cerebral cortex

**DOI:** 10.3389/fnana.2022.1035420

**Published:** 2022-11-10

**Authors:** Richard Jarrett Rushmore, Sylvain Bouix, Marek Kubicki, Yogesh Rathi, Edward Yeterian, Nikos Makris

**Affiliations:** ^1^Department of Anatomy and Neurobiology, Boston University School of Medicine, Boston, MA, United States; ^2^Psychiatry Neuroimaging Laboratory, Brigham and Women’s Hospital, Boston, MA, United States; ^3^Center for Morphometric Analysis, Massachusetts General Hospital, Boston, MA, United States; ^4^Department of Software Engineering and Information Technology, École de Technologie Supérieure, Montreal, QC, Canada; ^5^Department of Psychology, Colby College, Waterville, ME, United States

**Keywords:** MRI, cerebral cortex, atlas, Harvard-Oxford Atlas, macaque, cortical parcellation

## Abstract

Comparative structural neuroanatomy is a cornerstone for understanding human brain structure and function. A parcellation framework that relates systematically to fundamental principles of histological organization is an essential step in generating structural comparisons between species. In the present investigation, we developed a comparative parcellation reasoning system (ComPaRe), which is a formal ontological system in human and non-human primate brains based on the cortical cytoarchitectonic mapping used for both species as detailed by Brodmann. ComPaRe provides a theoretical foundation for mapping neural systems in humans and other species using neuroimaging. Based on this approach, we revised the methodology of the original Harvard-Oxford Atlas (HOA) system of brain parcellation to produce a comparative framework for the human (hHOA) and the rhesus monkey (mHOA) brains, which we refer to as HOA2.0-ComPaRe. In addition, we used dedicated segmentation software in the publicly available 3D Slicer platform to parcellate an individual human and rhesus monkey brain. This method produces quantitative morphometric parcellations in the individual brains. Based on these parcellations we created a representative template and 3D brain atlas for the two species, each based on a single subject. Thus, HOA2.0-ComPaRe provides a theoretical foundation for mapping neural systems in humans and other species using neuroimaging, while also representing a significant revision of the original human and macaque monkey HOA parcellation schemas. The methodology and atlases presented here can be used in basic and clinical neuroimaging for morphometric (volumetric) analysis, further generation of atlases, as well as localization of function and structural lesions.

## Introduction

Brain function and behavior are derived from the complex interrelations among connected networks of neural systems (e.g., Mesulam, 1985, 2000; Pandya and Yeterian, 1985; Schmahmann and Pandya, 2006; Swanson, 2012; Pandya et al., 2015). Each brain region, through its connections, is positioned in one or more brain networks, thus playing a unique role in network function and specific aspects of behavior. The degree to which the brain regions comprising these networks contribute to normal or impaired function is a topic of intensive study in neuroscience. A refinement of the ability to identify specific brain regions in human neuroimaging is key to a more sophisticated understanding of normal brain function as well as the ways in which neurological and psychiatric diseases affect neural systems.

The analysis of brain structure in neuroimaging depends on the state of technology used to obtain and analyze brain images as well as the methods used to divide the brain into its component regions (e.g., Glasser et al., 2016; Van Essen and Glasser, 2018). In the cerebral cortex, the method of dividing regions of the cortical mantle into more discrete areas is known as cortical parcellation (e.g., Jouandet et al., 1989; Rademacher et al., 1992). The categorization of different cortical regions and areas was originally performed in post-mortem tissue using brain sectioning and histological techniques to visualize cell bodies or myelin (e.g., Brodmann, 1909; von Economo, 1927; Garey, 2006; Nieuwenhuys et al., 2008; Nieuwenhuys, 2013; Triarhou, 2013, 2020; Amunts and Zilles, 2015; Nieuwenhuys and Broere, 2020). In neuroimaging, parcellation of cerebral cortical areas at the level of microscopic precision is not yet possible. Thus, the precise relationships between the full range of discrete neuroanatomical areas and magnetic resonance imaging (MRI) images are not yet fully established (e.g., Elston and Garey, 2009, 2013; Amunts and Zilles, 2015; Glasser et al., 2016). In order to more precisely interrelate cortical areas as defined anatomically with those generated by structural neuroimaging methods to the extent allowed by MRI, a rationale and methodology is needed to parcellate cerebral cortical brain areas in a consistent manner (e.g., Kennedy et al., 1998; Caviness et al., 1999). To this end, the Center for Morphometric Analysis (CMA) at Massachusetts General Hospital (MGH) was the first to employ a self-referential approach using consistent anatomical landmarks (e.g., brain sulci and gyri) to establish a set of rules for demarcation and volumetric measurement of specific cerebral cortical areas in individual brains (Rademacher et al., 1992; Caviness et al., 1996). This system of analysis led to the creation of the original Harvard-Oxford Atlas (HOA), one of the earliest systematic frameworks for parcellating an individual human brain in the neuroimaging domain (Jenkinson et al., 2012). The HOA approach subsequently served as a foundation for other methods of fully automated brain parcellation (e.g., Fischl et al., 2002, 2004; Desikan et al., 2006; Klein et al., 2017).

The HOA parcellation system remains an important approach that needs to be updated in light of neuroscientific advances and improvements in non-invasive neuroimaging technology. The present investigation updates the HOA system of cerebral cortical brain parcellation (Frazier et al., 2005; Desikan et al., 2006; Makris et al., 2006a; Goldstein et al., 2007; Jenkinson et al., 2012) to include more regions that are relevant to structural, functional, and clinical neuroimaging studies. This update of the HOA, referred to as HOA2.0, includes both a revised human brain parcellation (hHOA2.0) and an update of the HOA system for the monkey (mHOA2.0) (Makris et al., 2010; Rushmore et al., 2020a) to relate macaque monkey and human brain structures. This approach is based on the reasoning that comparability between macaque and human cortical areas is guided by structural features such as cytoarchitecture and structural connectivity (e.g., Brodmann, 1909; Pandya and Yeterian, 1985; Garey, 2006). Thus, the methodology and atlases presented herein address the need for a finer-grained, histologically informed and MRI-based methodological framework for the human and the monkey brain.

In this study, we developed a morphometric methodology that can be applied to both monkey and human brains. We created parcellation frameworks in both species using the same software tools, and comparable ontologies and anatomical criteria. The resulting revised human and monkey atlases have been developed within this common HOA theoretical framework, which we term the HOA2.0-Comparative Parcellation Reasoning (HOA2.0-ComPaRe) system. The original HOA framework has thus been refined and expanded in light of current information from different neuroscientific disciplines. We expect the HOA2.0-ComPaRe system to provide a foundation for a more refined understanding of structural and functional neuroimaging studies in monkey and human brains.

## Methods

### Subjects

Magnetic resonance images were collected from a single 33-year-old Caucasian right-handed human male volunteer, and a single 7-year-old female rhesus monkey (*Macaca mulatta*), comparatively equivalent to a young adult human (approximately 20 years of age). For the human subject, written informed consent was obtained after a description of the study was given, and procedures were approved by the Institutional Review Board (IRB) at Massachusetts General Hospital. All animal procedures were approved by the Institutional Animal Care and Use Committees (IACUCs) at Boston University School of Medicine and Massachusetts General Hospital.

### Human subject

The MRI images for the human subject were acquired at the A. A. Martinos Center for Biomedical Imaging at Massachusetts General Hospital using a Siemens Trio 3T imaging system. Scans included a T1-weighted acquisition with the following parameters: TE = 3.3 ms, TR = 2,530 ms, TI = 1,100 ms, flip angle = 7°, slice thickness = 1.33 mm, 128 contiguous sagittal slices, acquisition matrix = 256 × 256, in-plane resolution = 191 mm^2^ (i.e., FOV = 256 mm × 256 mm), two averages and pixel bandwidth = 200 Hz/pixel. The total acquisition time was approximately 8 min.

### Monkey subject

MRI imaging for the monkey was performed under ketamine-xylazine anesthesia (20 mg/kg; 0.2–0.4 mg/kg). The monkey was placed in an MRI-compatible head holder and scanned in a 1.5T Siemens Sonata magnet at the MGH-NMR Center at the Charlestown Navy Yard. MP-RAGE volumes with 0.8 mm × 0.8 mm in-plane resolution and 1.0 mm thick slices were acquired using the following parameters: TR = 2.73 ms, TE = 2.8 ms, TI = 300 ms, flip angle = 7°, matrix = 256 × 256, bandwidth = 190 Hz/pixel, NEX = 4, with a total acquisition time of 40 min. Approximately 128 slices were acquired with zero gap, increasing slice thickness to cover the brain.

### Magnetic resonance imaging preprocessing

For both the human and the monkey brain, images were resampled into a standard coordinate system (Filipek et al., 1994; Makris et al., 2004). A new set of coronal images, not rescaled, was reconstructed at the slice thickness of the original acquisition. Neuroanatomic segmentation was performed on coronal images using semiautomated morphometric techniques (Filipek et al., 1994; Caviness et al., 1996; Worth et al., 1997; Makris et al., 2004, 2006b). The cerebrum was segmented into its principal gray matter and white matter structures and total cerebral white matter (Makris et al., 1997, 1999; Kennedy et al., 1998; Rushmore et al., 2020a). Specifically, the cortical ribbon was defined by two outlines, one external outline between the subarachnoid CSF and the cerebral cortex, and the other between the cerebral cortex and the underlying cerebral white matter (Worth et al., 1997; Makris et al., 2006b; Rushmore et al., 2020a). The total number of voxels in each brain region represented its volume.

### Surface generation

Using FreeSurfer on T1-weighted MRI datasets, the segmented volume of the cerebrum and cerebral white matter was converted into a surface representation. This conversion process was performed using a custom designed extension of the FreeSurfer environment (Fischl et al., 1999), in part implemented through the use of TKMedit and TKSurfer programs (Pienaar et al., 2020). The inflated white matter surface was used in both brains to identify and trace sulci and anatomical planes, which serve as the borders for parcellation units. Parcellation units were imported into 3D Slicer for visualization and volumetric analysis. It should be noted that the terms sulcus (*pl* sulci) and fissure (*pl* fissures) are used interchangeably in the present study.

### Principles of the HOA2.0-ComPaRe system

The HOA2.0-ComPaRe system incorporates both ontology and comparative neuroanatomy. Ontology in the present neuroanatomical context consists of names corresponding to the operationally defined brain structures they represent (adapted from Bowden and Dubach, 2003; D. Bowden, personal communication). Comparative structural neuroanatomy is based on correspondences such as cellular composition and anatomical position of brain regions between different species.

Morphometric analysis using neuroimaging raises an ontological question of how cortical regions of interest (ROIs) correspond between species. For instance, how does a specific ROI such as the anterior cingulate gyrus in the monkey relate to a similar structure in the human brain? Such relationships have been established by classical neuroanatomists such as Brodmann (Brodmann, 1909; Garey, 2006), and Bailey and von Bonin (1951). In Brodmann’s framework, cytoarchitectonic areas were demarcated on the basis of structural criteria across several species and found to be comparable. Thus, for example, the anterior cingulate gyrus is characterized by a specific laminar and cellular composition in both macaque and human brains and labeled in both as Brodmann area 24. Cytoarchitectonic analysis was performed by Brodmann across cerebral cortical regions to produce comparative maps in human and non-human primates. These cytoarchitectonically aligned maps continue to be used widely in basic and clinical neuroscientific research.

A comparative approach is crucial for translating experimental animal results to humans. For cerebral cortical anatomy and structural connectivity, correspondence between macaque and human brain structures allows for accurate translation of findings (e.g., Bowden and Martin, 1995; Bowden and Dubach, 2003; Bowden et al., 2012; Swanson, 2015; Rushmore et al., 2020b). We have recently discussed the importance of comparative approaches for our understanding of human brain neuroanatomy (Rushmore et al., 2020a,b). In the present study, we developed a comparative morphometric method for both monkey and human brains and created parcellation frameworks in both species using the same software tools, and comparable ontologies and anatomical criteria. The resulting human and monkey atlases have been developed within this common HOA2.0 framework, which we have termed the Comparative Parcellation Reasoning (ComPaRe) system.

### Human brain cortical parcellation

The method of cortical parcellation was based on that of Caviness et al. (1996), which constitutes the basis of the original HOA, a probabilistic human brain atlas included in the FSL software package (Jenkinson et al., 2012). The HOA as first detailed by Caviness et al. (1996) used coronal planes and limiting sulci to create 48 parcellation units. In the present report, this schema has been expanded to produce a finer-grained parcellation based on a current understanding of cortical regions and areas (see below). This revised system, termed the human HOA2.0 (hHOA2.0) now includes 73 parcellation units (27 frontal lobe, 13 parietal lobe, 15 temporal lobe, 9 occipital lobe, 7 paralimbic, 2 insular). The additional parcellation units in the hHOA2.0 are made up of subdivisions of the original parcellation units. Modifications have been made to the frontal, parietal and occipital lobes and the insula, while temporal lobe and limbic lobe parcellation units remain unchanged. The modifications are summarized below.

The frontal pole parcellation unit (PU) was defined by Caviness et al. (1996) as the cortex anterior to a coronal plane positioned at the tip of the anterior horizontal ramus of the Sylvian fissure. The resulting parcellation unit included much of the anterior portions of the superior, middle and inferior frontal gyri. By repositioning the coronal plane specifying the posterior limit of the frontal pole to the anterior terminus of the olfactory sulcus, the frontal pole PU was reduced in extent to better approximate Brodmann’s area 10 (Brodmann, 1909; Garey, 2006; Bludau et al., 2014). This modification allows subparcellation of the superior and middle frontal gyri into three main portions (anterior, middle, and posterior), and also enables subdivision of the anterior portion of the inferior frontal gyrus. These subdivisions are more consistent with the locations of Brodmann areas on these three gyri (Brodmann, 1909; Garey, 2006). A second consequence of modifying the posterior border of the frontal pole PU is that the orbital frontal cortex could be subdivided in accord with morphological divisions (e.g., Chiavaras and Petrides, 2000; Chiavaras et al., 2001; Ongür et al., 2003).

The frontal pole was further separated into medial and lateral components by the hemispheric margin, in line with neuroanatomical studies of this region (Brodmann, 1909; Garey, 2006; Bludau et al., 2014). The hemispheric margin also served to separate mesial and dorsolateral components of the superior frontal gyrus, and to specify the superior border of a novel pre-supplementary motor area (preSMA) PU. The borders of this region were determined with reference to studies of cytoarchitecture and function (Zilles et al., 1996; Vorobiev et al., 1998; Kim et al., 2010; Ruan et al., 2018), which showed that a coronal plane positioned on the anterior commissure divided cytoarchitectonic regions of the preSMA from the SMA. The preSMA was further divided into superior and inferior regions by the paracingulate sulcus to separate the paralimbic inferior component from the frontal superior component, a division important for mapping and targeting of preSMA using transcranial magnetic stimulation (TMS).

The precentral and postcentral gyri were each subdivided into four component regions based on knowledge of somatotopic organization. Mesial portions of the pre- and post-central gyri, which contain representations of the leg, have been separated from the lateral regions of the gyri by the hemispheric margin. The lateral gyral surfaces were further subdivided based on the presence of the omega signs in the pre- and post-central gyri, which constitute the morphological analogs of the motoric and somatosensory hand representations, respectively (Rasmussen and Penfield, 1947; White et al., 1997; Yousry et al., 1997; Moore et al., 2000; Blankenburg et al., 2003; van Westen et al., 2004; Nelson and Chen, 2008; Hong et al., 2018; Dalamagkas et al., 2020). The regions defined by the omega signs comprise the middle subdivisions of the pre- and post-central gyrus PUs and as a result define superior and inferior divisions for each gyrus. On the opercular surface of these gyri, the central operculum PU was divided into anterior and posterior regions, the separation of which was defined by a plane through the inferior margin of the central sulcus. More inferiorly, the insula was subparcellated into anterior and posterior segments by the central sulcus of the insula (Makris et al., 2006a; Kurth et al., 2010; Evrard, 2019).

The posterior parietal cortex was previously separated by Caviness et al. (1996) into superior parietal lobule, angular gyrus and supramarginal gyrus parcellation units. In the current parcellation schema, the angular gyrus was divided into anterior and posterior portions based on structural and functional grounds (Caspers et al., 2006, 2008; Uddin et al., 2010; Cieslik et al., 2013) and the superior parietal lobule was divided into anterior and posterior regions to better reflect cytoarchitectonic divisions (Brodmann, 1909; Garey, 2006; Scheperjans et al., 2008).

The occipital lobe previously contained a parcellation unit comprising both banks of the calcarine sulcus (Caviness et al., 1996). This parcellation unit was subdivided into superior and inferior divisions to reflect the differing retinotopy of the two sulcal banks (see Glickstein, 1988 for review).

### Macaque brain cortical parcellation

The original cortical parcellation of the macaque was based on the Harvard Oxford Atlas and referred to as the macaque HOA (mHOA) (Makris et al., 2010; Rushmore et al., 2020a). This parcellation contained 26 PUs. In the current refined version, the mHOA2.0 is more closely aligned with the modified schema for the human brain, as detailed above. The modified mHOA2.0 now comprises 40 PUs (16 frontal lobe, 7 parietal lobe, 5 temporal lobe, 7 occipital lobe, 4 paralimbic, 1 insular). As in the human, modifications were made in the frontal, parietal and occipital lobes, whereas parcellation units in the temporal lobe, limbic lobe and insula were not modified.

In the frontal lobe, a tripartite prefrontal gyral organization was introduced (e.g., Bowden and Martin, 1995) such that the cortex between the hemispheric margin and the sulcus principalis was divided into two parts based on an anterior extension of the superior limb of the arcuate sulcus. The precentral gyrus PU, which previously extended from the central sulcus posteriorly to the arcuate sulcus anteriorly, was subdivided into two premotor regions (dorsal and ventral) anterior to a coronal plane through the anterior commissure, with the precentral gyrus PU now referred to as the cortex between the central sulcus and the coronal plane defined by the anterior commissure. On the mesial frontal lobe surface, a novel preSMA region was extracted from the original PRG PU. Since the division between the SMA and the medial PRG could not be ascertained with certainty, these two regions were combined into a more caudal medial PRG/SMA PU.

The orbital surface of the frontal lobe, previously defined as a single PU, was now subdivided with reference to comparative anatomical studies of the orbitofrontal cortex (Chiavaras and Petrides, 2000; Chiavaras et al., 2001) into five PUs (FOCa, FOCm, FOCL, FOCp, FMC) that parallel those detailed above for the human orbital cortex.

In the parietal lobe, the postcentral gyrus PU was subdivided into medial and lateral portions based on the hemispheric margin. The temporal lobe opercular surface was subdivided into anterior and posterior supratemporal plane PUs to reflect the organization of the human temporal opercular region.

In the occipital lobe, the superior and inferior calcarine banks were delineated to reflect the function and anatomy of the calcarine sulcus in the human brain. In addition, the dorsolateral striate cortex above the calcarine sulcus was subdivided into superior and inferior portions by the presence of the ectocalcarine sulcus.

### Segmentation volumes

Once the PUs were defined in the monkey and the human brain, the volumes of each PU were derived by converting the representation on the white matter and pial surfaces to a volumetric space.

### Parcellation unit visualization

Parcellation units in both species were visualized by illustrating the borders of each parcellation unit on brain surfaces overlaid with the curve scalar (Makris et al., 2006b, 2008a). This permits a conjunctive viewing of anatomical and PU borders.

## Results

In this study, we developed a comparative methodology to parcellate brain structures in the monkey and the human brains, updated the theoretical framework underlying this methodology to include a more comprehensive set of brain structures based on accrued neuroscientific knowledge, and produced a representative template brain atlas for each species. To achieve a comparative framework, the macaque and human HOA2.0 systems were aligned using a methodology that allows the parcellation of any individual brain in humans and monkeys. This methodology is based on a common neuroanatomical method and framework, and implemented in the same software platform, specifically 3D Slicer (Fedorov et al., 2012). The parcellations for monkey and human cerebral cortical areas were updated to include more fine-grained regions of interest (ROIs), or parcellation units (PUs).

### Human cortical parcellation

We expanded the original human HOA framework as generated by Rademacher et al. (1992) and revised by Caviness et al. (1996). This framework apportions the cerebral cortex into parcellation units (PUs) that are defined by anatomical landmarks, cerebral sulci, and coronal limiting planes. In the present revision of this parcellation system, we identified regions within the original PUs that have been demonstrated to be distinct on structural or functional grounds. The PUs that comprise this system are identified in Table 1, with the modified PUs in bold. The anatomical landmarks and limiting planes are identified in Table 2, and the sulci used in the system are abbreviated in Table 3. Table 3 also specifies the relationship of each sulcus to established ontological entities, namely Neuronames (Bowden et al., 2012) Terminologica Neuroanatomica and FIPAT (Ten Donkelaar et al., 2017).

**TABLE 1:** Parcellation units—Human HOA (hHOA).

**AGa**	**Angular gyrus, anterior**
**AGp**	**Angular gyrus, posterior**
**CALCi**	**Intracalcarine cortex, inferior**
**CALCs**	**Intracalcarine cortex, superior**
**CGa_a**	**Cingulate gyrus, anterior, anterior part**
**CGa_p**	**Cingulate gyrus, anterior, posterior part**
CGp	Cingulate gyrus, posterior
CN	Cuneal cortex
**COa**	**Central opercular cortex, anterior**
**COp**	**Central opercular cortex, posterior**
**F1La**	**Superior frontal gyrus, lateral, anterior**
**F1Lm**	**Superior frontal gyrus, lateral, middle**
**F1Lp**	**Superior frontal gyrus, lateral, posterior**
**F1m**	**Superior frontal gyrus, medial**
**F2a**	**Middle frontal gyrus, anterior**
**F2m**	**Middle frontal gyrus, middle**
**F2p**	**Middle frontal gyrus, posterior**
**F3a**	**Inferior frontal gyrus, anterior**
F3o	Inferior frontal gyrus, pars opercularis
**F3orb**	**Inferior frontal gyrus, pars orbitalis**
F3t	Inferior frontal gyrus, pars triangularis
FMC	Frontal medial cortex
FO	Frontal opercular cortex
**FOCa**	**Frontal orbital cortex, anterior**
**FOCL**	**Frontal orbital cortex, lateral**
**FOCm**	**Frontal orbital cortex, medial**
**FOCp**	**Frontal orbital cortex, posterior**
**FPL**	**Frontal pole, lateral**
**FPm**	**Frontal pole, medial**
H1	Heschl’s gyrus
**INSa**	**Insular cortex, anterior**
**INSp**	**Insular cortex, posterior**
LG	Lingual gyrus
OF	Occipital fusiform gyrus
OLi	Lateral occipital cortex, inferior
OLs	Lateral occipital cortex, superior
OP	Occipital pole
PAC	Paracingulate gyrus
PCN	Precuneal cortex
PHa	Parahippocampal gyrus, anterior
PHp	Parahippocampal gyrus, posterior
PO	Parietal opercular cortex
**POGLi**	**Postcentral gyrus, lateral, inferior**
**POGLm**	**Postcentral gyrus, lateral, middle**
**POGLs**	**Postcentral gyrus, lateral, superior**
**POGm**	**Postcentral gyrus, medial**
PP	Planum polare
**PreSMAi**	**Pre-supplementary motor area, inferior**
**PreSMAs**	**Pre-supplementary motor area, superior**
**PRGLi**	**Precentral gyrus, lateral, inferior**
**PRGLm**	**Precentral gyrus, lateral, middle**
**PRGLs**	**Precentral gyrus, lateral, superior**
**PRGm**	**Precentral gyrus, medial**
PT	Planum temporale
SC	Subcallosal cortex
SCALC	Supracalcarine cortex*
SGa	Supramarginal gyrus, anterior
SGp	Supramarginal gyrus, posterior
SMA	Supplementary motor area
**SPLa**	**Superior parietal lobule, anterior**
**SPLp**	**Superior parietal lobule, posterior**
T1a	Superior temporal gyrus, anterior
T1p	Superior temporal gyrus, posterior
T2a	Middle temporal gyrus, anterior
T2p	Middle temporal gyrus, posterior
T3a	Inferior temporal gyrus, anterior
T3p	Inferior temporal gyrus, posterior
TFa	Temporal frontal cortex, anterior
TFp	Temporal frontal cortex, posterior
TO2	Middle temporal gyrus, temporo-occipital
TO3	Inferior temporal gyrus, temporo-occipital
TOF	Temporal occipital fusiform cortex
TP	Temporal pole

Modified parcellation units are denoted in bold text.

*Equivalent to the SCAL parcellation unit of Caviness et al. (1996).

**TABLE 2:** Human HOA (hHOA) anatomical landmarks.

BF	Orbitofrontal cortex, posterior limit
Plane A	Anterior horizontal ramus of Sylvian fissure, anterior limit
Plane B	Isthmus of temporal and frontal lobes
Plane C	Sulcus of Heschl, anterior limit
Plane D	Sylvian fissure, posterior limit
Plane E	Intermediate sulcus of Jensen, inferior limit
Plane F	Opercularization of the intraparietal sulcus
Plane G	Cuneal sulcus, posterior limit
Plane H	Paracingulate gyrus, anterior limit
Plane I	Corpus callosum, anterior limit
Plane J	Decussation of anterior commissure
Plane K	Precentral sulcus junction with hemispheric margin
Plane L	Central sulcus junction with hemispheric margin
Plane M	Lateral geniculate nucleus
Plane N	Calcarine sulcus, anterior limit
Plane O	Precentral sulcus junction with Sylvian fissure
Plane P	Postcentral sulcus junction with Sylvian fissure
Plane Q	Olfactory sulcus, anterior limit
Plane R	Central sulcus junction with Sylvian fissure
SEP	Subcallosal cortex, posterior limit

**TABLE 3:** Limiting sulci in the human Harvard-Oxford Atlas (hHOA).

Sulcus	Abbreviation	Latin	Other names	Neuronames	TNA2 ID	FIPAT ID
Anterior ascending ramus of lateral fissure	aar	Ramus ascendens sulci lateralis	Anterior ascending limb of lateral fissure	68	5,451	2005
Angular sulcus	ag					
Anterior horizontal ramus of lateral fissure	ahr	Ramus anterior sulci lateralis	Anterior ascending limb of lateral fissure	69	5,449	2006
Calcarine sulcus	calc	Sulcus calcarinus		44	5,486	2139
Callosal sulcus	ca	Sulcus corporis callosi	Sulcus of the corpus callosum	36	5,439	2083
Central sulcus	ce	Sulcus centralis		48	5,435	2088
Central sulcus of the insula	cei	Sulcus centralis insulae	Central insular sulcus	112	5,505	2078
Cingulate sulcus	ci	Sulcus cinguli		43	5,440	2084
Circular sulcus of the insula	cir	Sulcus circularis insulae	Limiting sulcus	51	5,444	2079
Collateral sulcus	co	Sulcus collateralis		47	5,442	2087
Cuneal sulcus**	cun	n.d.	Superior sagittal sulcus of cuneus	n.d.	n.d.	n.d.
First transverse sulcus**	ftr	Sulcus temporalis transversus anterior	Anterior transverse temporal sulcus	n.d.	n.d.	n.d.
Heschl’s sulcus**	He*	Sulcus temporalis transversus posterior	Posterior transverse temporal sulcus	n.d.	n.d.	n.d.
Hippocampal fissure	hi	Sulcus hippocampalis		42	5,522	2183
Inferior frontal sulcus	if	Sulcus frontalis inferior		63	5,453	2018
Inferior temporal sulcus	it	Sulcus temporalis inferior		130	5,496	2072
Intermediate sulcus of Jensen	im	Sulcus intermedius primus	Primary intermediate sulcus	2,382	n.d.	n.d.
Intraparietal sulcus	ip	Sulcus intraparietalis		97	5,475	2037
Lateral occipital sulcus	lo	n.d.		143	n.d.	n.d.
Lateral orbital sulcus	lorb*	Sulcus orbitalis lateralis		81	n.d.	2111
Marginal ramus of the cingulate sulcus						
Medial orbital sulcus	morb	Sulcus orbitalis medialis		82	n.d.	2113
Occipitotemporal sulcus	ot	Sulcus occipitotemporalis		55	5,438	2147
Olfactory sulcus	ol	Sulcus olfactorius		78	5,463	2115
Paracingulate sulcus	pa	n.d.		2,399	n.d.	n.d.
Parietooccipital sulcus	po	Sulcus parietooccipitalis		52	5,437	2007
Postcentral sulcus	pcs	Sulcus postcentralis		99	5,740	2035
Posterior ascending ramus of the Sylvian fissure						
Posterior horizontal ramus of the Sylvian fissure						
Precentral sulcus	prs	Sulcus precentralis		3,474	5,457	2027
Subparietal sulcus	sp	Sulcus subparietalis	Splenial sulcus	102	5,441	2135
Superior frontal sulcus	sf	Sulcus frontalis superior		61	5,455	2032
Superior temporal sulcus	st	Sulcus temporalis superior		129	5,494	2070
Transverse orbital sulcus	torb	Sulcus orbitalis transversus		80	n.d.	2112

*May exist as two or more sulci, which may be indicated by numerical suffix (e.g., Heschl’s sulcus 1, Heschl’s sulcus 2).

**Not present in Neuronames. n.d., not designated.

### Additional sulci

Several sulci were added to the original human and monkey HOA systems to enable parcellation unit subdivisions. In the orbitofrontal region, an H-shaped sulcal pattern was identified. The medial orbital sulcus and the lateral orbital sulcus form the vertical limbs of the H, and the transverse orbital sulcus forms the horizontal limb (Chiavaras and Petrides, 2000; Poellinger et al., 2001). Another sulcus added to the original hHOA system is the central sulcus of the insula, which separates the anterior long insular gyri from the posterior short insular gyri (Evrard, 2019).

### Additional anatomical landmarks

Several anatomical landmarks were added to the revised HOA system. The presence of the omega sign in the precentral gyrus, and its analog in the postcentral gyrus, provide a landmark for the hand motoric and sensory representations, respectively. Lines can be traced above and below these landmarks to divide the lateral aspects of the pre- and post-central gyri into three portions. The lines on the precentral gyrus are known as the superior and inferior precentral lines (SPRCL, IPRCL). Similarly, the lines on the postcentral gyrus are referred to as the superior and inferior postcentral lines (SPOCL, IPOCL). An additional line is designated in this system in the parietal lobe. This line, termed the parietal line (PAL), connects the preoccipital notch to the superior terminus of the parieto-occipital sulcus at the hemispheric margin (Supplementary Figure 1). This line separates posterior parietal and inferior temporal regions from occipital regions. Furthermore, two additional coronal planes were specified. Plane Q was placed at the anterior terminus of the olfactory sulcus and defines the posterior borders of the frontal pole PU on the ventral and lateral aspects of the hemisphere. Plane R was positioned at the inferior terminus of the central sulcus to divide the central operculum PU into anterior and posterior regions.

**FIGURE 1:**
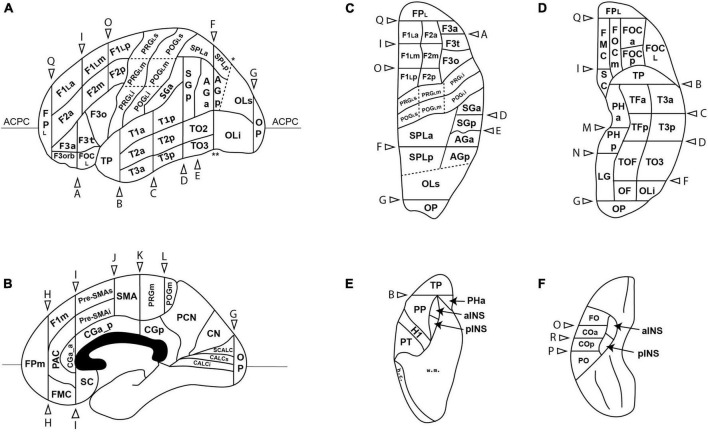
Schematic diagram of the revised Harvard-Oxford Atlas parcellation of the human brain, hHOA2.0, from the lateral **(A)**, mid-sagittal **(B)**, superior **(C)** and inferior **(D)** perspectives. The opercular and insular surfaces of the Sylvian fissure are depicted from superior **(E)** and inferior **(F)** views that show the temporal opercular surfaces **(E)** and the frontal and parietal opercular surfaces **(F)**. Solid lines indicated by letters correspond to limiting planes. Curved lines represent sulci, and dotted lines represent non-sulcal or plane PU borders. The single asterisk in A is the superior location of the parieto-occipital sulcus, and the double asterisk is the pre-occipital notch. ACPC, anterior posterior plane; h.c., hemispheric convexity of parietal lobe; w.m., subcortical white matter. All other abbreviations are listed in Table 1. Figure modified from Caviness et al. (1996).

**FIGURE 2:**
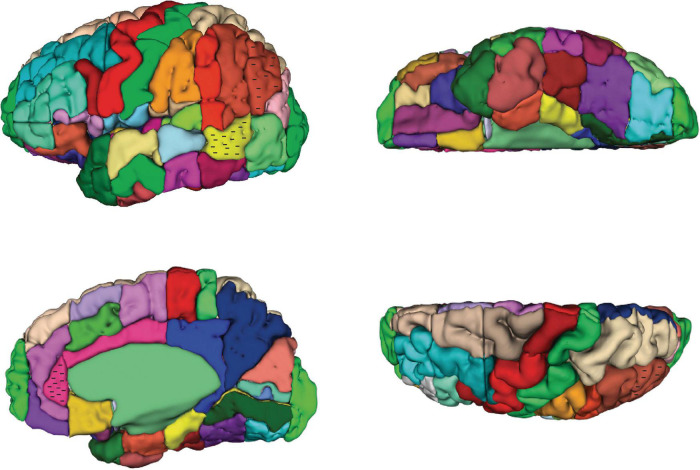
Three-dimensional representation of the revised Harvard-Oxford Atlas parcellation of the human brain, hHOA2.0. Refer to Figure 1 for parcellation unit (PU) identity.

The anatomical landmarks, planes and sulci together define the borders for each parcellation unit as defined in Table 4 and visualized in Figures 1, 2.

**TABLE 4:** Human Harvard-Oxford Atlas (hHOA) parcellation unit definitions.

PU	Ant	Post	Med/Inf	Lat/Sup
AGa	CP E, im	CP F	ip	st, lo
AGp	CP F	PAL	ip	st, lo
CALCi	po	CP G	calc	CIL
CALCs	po	CP G	CSL	calc
CGa_a	ci	CP I	ci	ci
CGa_p	CP I	CP K	ca	ci
CGp	CP K	sp	ca, calc	ci, sp
CN	po	CP G	cun	HM
COa	CP O	CP R	cir	S45D
COp	CP R	CP P	cir	S45D
F1La	CP Q	CP I	HM	sf
F1Lm	CP I	CP O	HM	sf
F1Lp	CP O	prc	HM	sf
F1m	CP H	CP I	pa	HM
F2a	CP Q	CP I	sf	if
F2m	CP I	CP O	sf	if
F2p	CP O	prc	sf	if
F3a	CP Q	CP A	if	ahr
F3o	aar	prc	if	S45D
F3orb	CP Q	CP A	lorb*	S45/ahr
F3t	CP A	aar	if	S45D
FMC	CP Q	CP I	pa	ol
FO	n/a	CP O	cir	S45D
FOCa	CP Q	torb	lorb	morb
FOCL	CP Q	circ	lorb	S45D/lorb*
FOCm	CP Q	BF	morb	ol
FOCp	torb	BF, circ	morb	lorb
FPL	HM	CP Q	HM	HM
FPm	HM	CP H	HM	HM
H1	I45D	circ	ftr	He
INSa	circ	cei	circ	circ
INSp	cei	circ	circ	circ
LG	CP N	CP G	calc	co
OF	CP F	CP G	co	ot
OLi	CP F	CP G	ot	lo
OLs	PAL	CP G	HM	lo
OP	CP G	HM	HM	HM
PAC	pa	CP I, Ci	pa	pa
PCN	sp	po	calc	HM
PHa	CP B	CP M	co	hi
PHp	CP M	CP N	co	hi, calc
PO	CP P	CP D	cir	S45D
POGLi	ce	poc	S45D	IPOCL
POGLm	ce	poc	IPOCL	SPOCL
POGLs	ce	poc	SPOCL	HM
POGm	CP L	ci	ci	HM
PP	CP B	ftr	circ	I45D
PreSMAi	CP I	CP J	ci	pa
PreSMAs	CP I	CPJ	pa	HM
PRGLi	prc	ce	S45D	IPRCL
PRGLm	prc	ce	IPRCL	SPRCL
PRGLs	prc	ce	SPRCL	HM
PRGm	CP K	CP L	ci	HM
PT	He	CP D	circ	I45D
SC	CP I	BF, SEP	ol	ca
SCALC	poc	CP G	CSL	cun
SGa	poc	CP D	ip	S45D
SGp	CP D	CP E, im	ip	st
SMA	CP J	CP K	ci	HM
SPLa	poc	CP F	ip	HM
SPLp	CP F	PAL	ip	HM
T1a	CP B	CP C	I45D	st
T1p	CP C	CP D	I45D	st
T2a	CP B	CP C	st	it
T2p	CP C	CP D	st	it
T3a	CP B	CP C	it	ot
T3p	CP C	CP D	it	ot
TFa	CP B	CP C	ot	co
TFp	CP C	CP D	ot	co
TO2	CP D	CP F	st, lo	it
TO3	CP D	CP F	it	ot
TOF	CP D	CP F	ot	co
TP	HM	CP B	HM	HM

Abbreviations are found in Table 1 for parcellation units, and Table 3 for sulci. Coronal planes (CP) are listed in Table 2. CIL, calcarine inferior line; CSL, calcarine superior line; HM, hemispheric margin; I45D, inferior 45 degree line of the Sylvian fissure; S45D, superior 45 degree line of the Sylvian fissure; IPOCL, inferior postcentral line; IPRCL, inferior precentral line; SCL, superior calcarine line; SPOCL, superior postcentral line; SPRCL, superior precentral line.

*In cases where the lateral orbital sulcus is duplicated, the lateral FOC PU is divided from the F3o PU based on this sulcus. In cases where such a sulcus is not identified, the two PUs are combined into a single PU, denoted as FOCL/F3o.

### Novel parcellation units—Frontal lobe

In the frontal lobe, most major gyri of the original HOA were subdivided further. As indicated above, the precentral gyrus was first divided into medial (PRGm) and lateral portions by the hemispheric margin. The lateral portion was then subdivided into three parts (PRGLi, PRGLm, PRGLs) based on the presence of the omega sign. The superior frontal gyrus, termed the F1 PU by Caviness et al. (1996), was similarly subdivided into medial and lateral portions. The medial portion was then separated into an F1m portion, and into SMA and preSMA regions. The lateral portion of F1 was further subdivided by existing limiting planes into anterior (F1La), middle (F1Lm) and posterior (F1Lp) portions. A similar partition was performed in the middle frontal gyrus (F2) to create anterior, middle and posterior F2 PUs (F2a, F2m, F2p). The inferior frontal gyrus, which previously contained two PUs (F3o, F3t), was expanded to include a total of four PUs with the addition of an anterior F3 (F3a) and an orbital F3 (F3orb) PU. As detailed above, the fronto-orbital PU was subdivided into 4 parts based on the H-shaped orbital sulcus: the anterior (FOCa), middle (FOCm), lateral (FOCL), and posterior (FOCp) fronto-orbital PUs. Finally, the frontal pole PU was subdivided into lateral (FPL) and medial (FPm) portions.

### Novel parcellation units—Parietal lobe

The postcentral gyrus was separated into medial and lateral portions based on the hemispheric margin. The lateral postcentral gyrus was subdivided into inferior, middle and superior PUs (POGLi, POGLm, POGLs) with reference to the postcentral gyrus equivalent of the omega sign. In addition, the central opercular cortex (CO) PU flanking the inferior terminus of the central sulcus, and originally spanning parietal and frontal regions, was subdivided into anterior (COa) and posterior (COp) PUs. The angular gyrus PU and the superior parietal lobule PU were both subdivided into anterior and posterior portions (AGa, AGp, SPLa, SPLp).

### Novel parcellation units—The insula

The central sulcus of the insula was used to divide the insular cortex into anterior (INSa) and posterior (INSp) PUs.

### Novel parcellation units—Occipital lobe

The superior and inferior banks of the calcarine sulcus (CALCs, CALCi) were specified as novel PUs.

### Structure-function relationships of human HOA2.0 parcellation units

Useful distinctions between and among brain areas can be made by considering the types of functions associated

with specific structurally defined areas (Mesulam, 1985, 2000; Rademacher et al., 1992). A general distinction between functional cerebral cortical types can be made according to whether the component regions are primary cortices (e.g., visual, auditory, motor, somatosensory), unimodal association cortices, heteromodal association cortices, or paralimbic association cortices. The relationship between each parcellation unit and its associated functional type is detailed in Supplementary Table 1.

### Morphometric analysis

Volumes for the hHOA2.0 parcellation units as delineated in the present study are listed in Table 5.

**TABLE 5:** Human Harvard-Oxford Atlas (hHOA) parcellation unit volumes from single subject.

PU	Right (cm^3^)	Left (cm^3^)
AGa	10.80	12.78
AGp	8.24	8.56
CALCi	1.77	2.04
CALCs	1.10	1.84
CGa_a	1.65	2.12
CGa_p	5.46	3.56
CGp	4.47	6.72
CN	4.52	4.86
COa	2.83	1.57
COp	1.34	2.89
F1La	5.09	2.17
F1Lm	4.75	5.70
F1Lp	8.91	6.16
F1m	2.13	1.82
F2a	6.67	8.69
F2m	8.38	9.37
F2p	4.61	2.98
F3a	5.08	4.71
F3o	3.87	4.42
F3orb	6.35	2.26
F3t	4.46	5.32
FMC	2.28	2.17
FO	2.79	5.02
FOCa	2.22	1.55
FOCL	6.23	3.74
FOCm	3.22	4.13
FOCp	2.60	4.07
FPL	6.47	7.36
FPm	4.85	2.23
H1	2.45	2.21
INSa	5.06	5.92
INSp	3.00	3.01
LG	5.17	5.10
OF	5.09	5.02
OLi	7.33	7.49
OLs	6.72	6.06
OP	19.03	9.04
PAC	4.82	5.44
PCN	14.21	13.31
PHa	2.86	3.08
PHp	1.94	2.59
PO	3.60	6.00
POGLi	4.51	5.09
POGLm	6.54	5.46
POGLs	3.97	3.66
POGm	3.41	2.57
PP	2.01	3.36
PreSMAi	3.39	4.54
PreSMAs	1.38	1.97
PRGLi	5.70	7.48
PRGLm	4.10	4.26
PRGLs	2.34	3.03
PRGm	2.97	3.13
PT	1.92	1.11
SC	2.91	3.19
SCALC	1.49	1.43
SGa	7.28	9.70
SGp	6.02	7.65
SMA	3.86	4.28
SPLa	9.70	11.99
SPLp	5.51	5.63
T1a	3.48	2.56
T1p	3.11	2.49
T2a	6.55	4.25
T2p	5.31	2.91
T3a	4.81	3.72
T3p	2.71	3.86
TFa	3.61	6.56
TFp	1.70	4.50
TO2	6.27	5.57
TO3	5.69	5.33
TOF	4.73	6.60
TP	7.87	10.19

#### Rhesus monkey cortical parcellation

**FIGURE 3:**
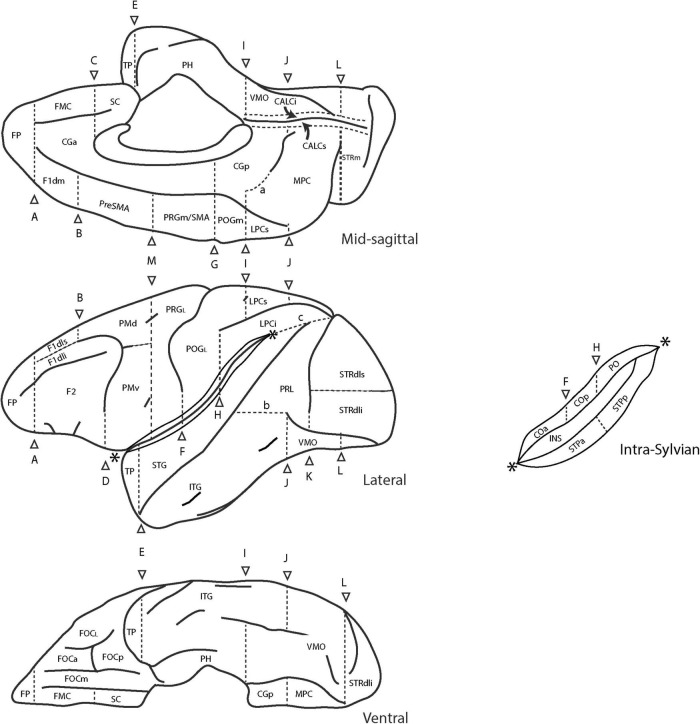
Schematic diagram of the revised Harvard-Oxford Atlas parcellation of the macaque monkey brain, mHOA2.0. Sulci are depicted as solid lines and limiting planes as dotted lines. Abbreviations are listed in Table 6. Figure modified from Rushmore et al. (2020a).

**FIGURE 4:**
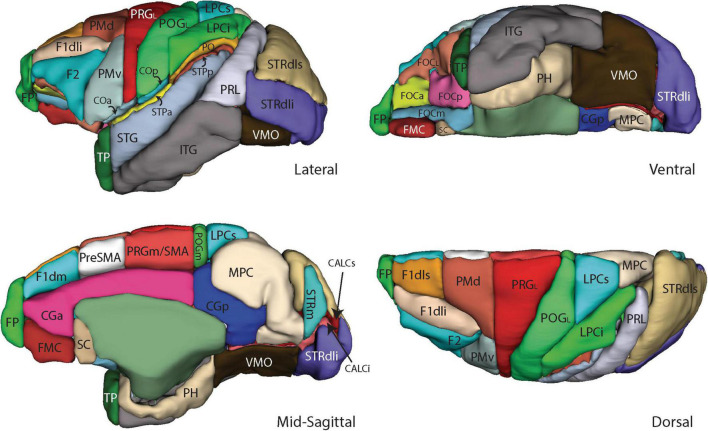
Three-dimensional representation of the revised Harvard-Oxford Atlas parcellation of the macaque monkey brain, mHOA2.0. Abbreviations are listed in Table 6.

The original extension of the HOA system of brain parcellation to the macaque monkey brain (Rushmore et al., 2020a) allowed for the division of the monkey cerebral cortex into parcellation units using the same methodological approach as in the human. Accordingly, PUs were defined on the basis of anatomical landmarks and borders visible and reliably identifiable using MRI. Table 6 lists these original PU abbreviations and shows the PUs of the present mHOA2.0 revision in bold. Anatomical landmarks are listed in Table 7, and sulci in Table 8. When applicable, sulcal abbreviations have been modified to parallel those used in the hHOA2.0. The PU definitions are listed in Table 9 and the cortical parcellation schema is illustrated in outline form in Figure 3 and in three dimensions in Figure 4.

**TABLE 6:** Parcellation units—macaque HOA (mHOA).

**CALCi**	**Intracalcarine cortex, inferior**
**CALCs**	**Intracalcarine cortex, superior**
CGa	Cingulate gyrus, anterior
CGp	Cingulate gyrus, posterior
COa	Central opercular cortex, anterior
COp	Central opercular cortex, posterior
**F1dli**	**Middle frontal gyrus**
F1dls	Superior frontal gyrus
F1dm	Superior frontal gyrus, medial
F2	Inferior frontal gyrus
**FMC**	**Frontal medial cortex**
**FOCa**	**Frontal orbital cortex, anterior**
**FOCL**	**Frontal orbital cortex, lateral**
**FOCm**	**Frontal orbital cortex, medial**
**FOCp**	**Frontal orbital cortex, posterior**
FP	Frontal pole
INS	Insular cortex
ITG	Inferior temporal gyrus
LPCi	Lateral parietal cortex, inferior
LPCs	Lateral parietal cortex, superior
MPC	Medial parietal cortex
PH	Parahippocampal gyrus
PO	Parietal opercular cortex
**POGL**	**Postcentral gyrus, lateral**
**POGm**	**Postcentral gyrus, medial**
**PMd**	**Premotor cortex, dorsal**
**PMv**	**Premotor cortex, ventral**
**PreSMA**	**Pre-supplementary motor area**
**PRGL**	**Precentral gyrus, lateral**
**PRGm/SMA**	**Precentral gyrus, medial/supplementary motor area**
PRL	Prelunate gyrus
SC	Subcallosal cortex
STG	Superior temporal gyrus
**STPa**	**Supratemporal plane, anterior**
**STPp**	**Supratemporal plane, posterior**
**STRdli**	**Striate cortex, dorsolateral, inferior**
**STRdls**	**Striate cortex, dorsolateral, superior**
STRm	Striate cortex, medial
TP	Temporal pole
VMO	Ventromedial occipital cortex

Modified parcellation units are denoted in bold text.

**TABLE 7:** Macaque HOA (mHOA) anatomical landmarks.

BF	Orbitofrontal cortex, posterior limit
Plane A	Rostral sulcus, anterior limit
Plane B	Superior ramus of arcuate sulcus, anterior limit
Plane C	Corpus callosum, anterior limit
Plane D	Inferior ramus of arcuate sulcus, inferior limit
Plane E	Isthmus of temporal and frontal lobes
Plane F	Central sulcus junction with Sylvian fissure
Plane G	Central sulcus junction with hemispheric margin
Plane H	Intraparietal sulcus, anterior limit
Plane I	Calcarine sulcus, anterior limit
Plane J	Inferior occipital sulcus, anterior limit
Plane K	Lunate sulcus, inferior limit
Plane L	Parietooccipital sulcus, inferior limit
Plane M	Decussation of anterior commissure
Plane a	Between superior limit of subparietal sulcus and coronal plane I
Plane b	Horizontal line from anterior limit of inferior occipital sulcus to superior temporal sulcus
Plane c	Between superior limit of Sylvian fissure, and confluence of lunate and intraparietal sulci

**TABLE 8:** Limiting Sulci in the macaque Harvard-Oxford Atlas (mHOA).

Sulcus	Neuronames abbreviation	Latin	Neuronames ID
Arcuate sulcus	arc	Sulcus arcuatus	2,379
Calcarine sulcus	ccs	Sulcus calcarinus	44
Callosal sulcus	cas	Sulcus corporis callosi	36
Central sulcus	ce	Sulcus centralis	48
Cingulate sulcus	cgs	Sulcus cinguli	43
External calcarine sulcus	ecs		146
Inferior calcarine sulcus	iccs		148
Inferior occipital sulcus	iocs		144
Intraparietal sulcus	itps	Sulcus intraparietalis	97
Lateral fissure	lf	Sulcus lateralis	49
Lateral orbital sulcus	los		81
Limiting sulcus of the insula	crs	Sulcus circularis insulae	51
Lunate sulcus	lus		150
Marginal sulcus	ms		98
Medial orbital sulcus	mos		82
Occipitotemporal sulcus	ots		55
Olfactory sulcus	olfs	Sulcus olfactorius	78
Parietooccipital sulcus	pos		52
Principal sulcus	prs	Sulcus principalis	66
Rhinal sulcus	rhs	Sulcus rhinalis	41
Rostral sulcus	ros		76
Subparietal sulcus	sbps		102
Superior calcarine sulcus	sccs		147
Superior temporal sulcus	sts		129
Transverse orbital sulcus	tos		80

**TABLE 9:** Macaque Harvard-Oxford Atlas (mHOA) parcellation unit definitions.

PU	Superior	Inferior	Anterior	Posterior
CALCi	ccs	CIL	ccs (ant)	ccs (post)
CALCs	CSL	ccs	ccs (ant)	ccs (post)
CGa	cgs	ros, cas	CP A	CP C, CP G
CGp	cgs	cas, ccs	CP G, CP I	CP I, CP J, Pl a, sbps
COa	HM	crs	Ant end insula	CP F
COp	HM	crs	CP F	Post end insula
F1dli	ASL	prs	CP A	arc
F1dls	HM	ASL	CP A	CP B
F1dm	HM	cgs	CP A	CP B
F2	prs	HM	CP A	CP D
FMC	ros	olfs	CP A	CP C
FOCa	los	mos	CP A	tos
FOCL	HM	los	CP A	BF
FOCm	mos	olfs	CP A	BF
FOCp	los	mos	tos	BF
FP	HM	HM	HM	CP A
INS	crs	crs	Ant end insula	Post end insula
ITG	sts, Pl b	ots, rhs	CP E	CP J
LPCi	ips	ls, Pl c	CP H	IPS, Pl c
LPCs	cgs	itps	CP I	CP J
MPC	ips, cfs	CSL	sbps, CP J, CP I, Pl a	CP L
PH	HPC	ots, rhs	CP E, HM	CP I
PO	HM	crs, ls	CP H	ls (post end)
POGL	HM	lf	ce, CP F	CP H, CP I
POGm	HM	cgs	CP G	CP I
PMd	HM	arc, APL	CP B	CP M
PMv	APL	HM, ls	arc, CP D	CP M
PreSMA	HM	cgs	CP B	CP M
PRGL	HM	ls	CP M	ce, CP F
PRGm/SMA	HM	cgs	CP M	CP G
PRL	Pl c	iocs, Pl b	sts	lus, CP K
SC	cas	olfs	CP C	BF
STG	ls	sts, Pl c	CP E, M	sts
STPa	crs, ls	HM	CP E	1/2 STP*
STPp	crs, ls	HM	1/2 STP*	ls (post end)
STRdli	ecs	ios, CIL	lus, CP L, CP K, pos	HM
STRdls	sccs	ecs	lus, pos, CP L	HM
STRm	HM	CSL	CP L	HM
TP	HM	HM	HM	CP E
VMO	CIL	ios	CP J	CP L

PU abbreviations found in Table 6. Sulcal abbreviations found in Table 8. ASL, superior arcuate line; HM, hemispheric margin; APL, posterior arcuate line; CP, coronal plane; Pl, plane; HPC, hippocampal sulcus; ICL, CIL, calcarine inferior line; CSL, calcarine superior line.

* 1/2 STP denotes the geometric mean of the supratemporal plane.

### Additional sulci and parcellation units

In the present study, the fronto-orbital region of the macaque was divided as in the human based on a similar H-shaped pattern of orbital sulci (Chiavaras and Petrides, 2000). These sulci provide the basis by which the prior single fronto-orbital PU was separated into five subdivisions (FOCa, FOCL, FOCm, FOCp, and FMC). The dorsolateral frontal lobe PUs were revised to more closely follow the tripartite gyral organization of the human brain. More specifically, the F1dls parcellation unit was placed above the principal sulcus and separated from the F1dli PU by a line extending from the superior limb of the arcuate sulcus toward the frontal pole (Bowden and Martin, 1995). The F2 PU was placed below the principal sulcus. The PRG parcellation unit, which originally extended from the arcuate sulcus anteriorly to the central sulcus posteriorly, and from the hemispheric margin laterally to the cingulate sulcus on the mesial surface, was subdivided into five novel parcellation units. On the lateral surface, the refined PRGL PU was separated from the more anterior dorsal (PMd) and ventral (PMv) premotor PUs. The original PRG PU on the medial surface was separated into an anterior preSMA PU and a posterior PRGm/SMA PU. In the parietal lobe, the hemispheric margin separated the lateral postcentral gyrus PU (POGL) from the medial POGm PU on the lateral surface. The temporal opercular cortex was separated into anterior and posterior PUs (STPa, STPp) based on the geometric mean of the supratemporal plane. In the occipital lobe, the banks of the calcarine sulcus were separated into inferior and superior (CALCi, CALCs) portions, and the dorsolateral striate cortex was separated into inferior and superior portions (STRdli, STRdls).

### Structure-function relationships of macaque HOA2.0 parcellation units

As above, correspondences between PUs and functional regions in the macaque brain were specified and listed in Supplementary Table 2.

### Morphometric analysis

Volumes for the mHOA2.0 parcellation units are listed in Table 10.

**TABLE 10:** Macaque Harvard-Oxford Atlas (mHOA) parcellation unit volumes from single subject.

PU	Right (cm^3^)	Left (cm^3^)
CALCi	0.51	0.49
CALCs	0.72	0.72
CGa	0.72	0.80
CGp	0.40	0.45
COa	0.19	0.20
COp	0.19	0.20
F1dli	0.61	0.53
F1dls	0.29	0.28
F1dm	0.28	0.25
F2	0.58	0.65
FMC	0.19	0.23
FOCa	0.20	0.14
FOCL	0.35	0.31
FOCm	0.21	0.21
FOCp	0.29	0.35
FP	0.30	0.38
INS	0.45	0.47
ITG	1.75	1.71
LPCi	0.98	0.96
LPCs	0.62	0.61
MPC	1.06	1.14
PH	0.62	0.63
PO	0.36	0.34
POGL	0.75	0.79
POGm	0.10	0.10
PMd	0.55	0.60
PMv	0.62	0.56
PreSMA	0.27	0.28
PRGL	0.76	0.82
PRGm/SMA	0.44	0.44
PRL	0.91	0.92
SC	0.09	0.10
STG	1.38	1.33
STPa	0.34	0.33
STPp	0.27	0.31
STRdli	1.06	1.15
STRdls	1.32	1.44
STRm	0.13	0.15
TP	0.43	0.39
VMO	0.78	0.83

### Comparative relationships of the human HOA2.0 and monkey HOA2.0 parcellation systems

A primary goal of extending the original human HOA system to the monkey brain was to relate the latter more systematically to the human brain (Rushmore et al., 2020a). As shown in Table 11, such a comparison can now be made between the PUs of the hHOA2.0 and mHOA2.0.

**TABLE 11:** HOA-ComPaRe Equivalences between human (hHOA) and macaque (mHOA) Harvard-Oxford Atlases.

	PU	hHOA PU	Human Brodmann areas	mHOA PU	Monkey Brodmann areas	Monkey Walker areas
Frontal Lobe	COa	Central opercular cortex - anterior	43	COa	43*	n.d.
	F1La	Superior frontal gyrus, lateral, anterior	8, 9	F1dls	9 (6, 8)	9, 8B
	F1Lm	Superior frontal gyrus, lateral, middle	8 (9, 6)	F1dls	9 (6, 8)	9, 8B
	F1Lp	Superior frontal gyrus, lateral, posterior	6	PMd	6	6
	F1m	Superior frontal gyrus, medial	8 (9)	F1dm	6 (32)	6, 8B, 9
	F2a	Middle frontal gyrus, anterior	46, 10 (9)	F1dli	9, 8 (10)	46 (8A)
	F2m	Middle frontal gyrus, middle	8, 9 (46)	F1dli	9, 8 (10)	46 (8A)
	F2p	Middle frontal gyrus, posterior	6	PMd	6	6
	F3a	Inferior frontal gyrus, anterior	10, 46	F2	10, 9, 8	12, 45, 46
	F3o	Inferior frontal gyrus, pars opercularis	44	F2	10, 9, 8	12, 45, 46
	F3orb	Inferior frontal gyrus, pars orbitalis	47	F2	10, 9, 8	12, 45, 46
	F3t	Inferior frontal gyrus, pars triangularis	45	F2	10, 9, 8	12, 45, 46
	FMC	Frontal medial cortex	11	FMC	9, 11, 12	14, 25 (10)
	FO	Frontal opercular cortex	44, 45	COa	n.d.**	n.d.
	FOCa	Frontal orbital cortex, anterior	11	FOCa	9, 10, 11, 12^†^	11 (10)
	FOCL	Frontal orbital cortex, lateral	11	FOCL	10 (11)^†^	12 (11)
	FOCm	Frontal orbital cortex, medial	11	FOCm	9, 11, 12^†^	14 (10)
	FOCp	Frontal orbital cortex, posterior	11	FOCp	11 (9)^†^	13^††^
	FPL	Frontal pole, lateral	10 (9, 11)	FP	9, 12	10
	FPm	Frontal pole, medial	10 (9, 11)	FP	9, 12	10
	PreSMAi	Pre-supplementary motor area, inferior	6 (medial)	PreSMA	4 (6)	n.d.
	PreSMAs	Pre-supplementary motor area, superior	6 (medial), 32	PreSMA	4 (6)	n.d.
	PRGLi	Precentral gyrus, lateral, inferior	4, 6	PRGL, PMv	4 (PRGL), 6 (PMv)	n.d.
	PRGLm	Precentral gyrus, lateral, middle	4, 6	PRGL	4	n.d.
	PRGLs	Precentral gyrus, lateral, superior	4 (6)	PRGL	4	n.d.
	PRGm	Precentral gyrus, medial	4 (medial)	PRGm/SMA	4 (3)	n.d.
	SMA	Supplementary motor area	4 (medial), 6 (medial)	PRGm/SMA	4, 3	n.d.
Occipital Lobe	CALCi	Intracalcarine cortex, inferior	17	CALCi	17	
	CALCs	Intracalcarine cortex, superior	17	CALCs	17	
	CN	Cuneal cortex	18, 19	STRm	17, 18 (19)	
	LG	Lingual gyrus	18, 19 (17)	VMO	19, 20 (18)	
	OF	Occipital fusiform gyrus	19	VMO	19, 20 (18)	
	OLi	Lateral occipital cortex, inferior	18, 19 (37)	VMO	19, 20 (18)	
	OLs	Lateral occipital cortex, superior	18, 19	PRL	18, 19	
	OP	Occipital pole	17, 18	STRdli, STRdls	17 (18)	
	SCALC	Supracalcarine cortex	17 (18)	STRm	17, 18	
Parietal Lobe	AGa	Angular gyrus, anterior	39 (anterior)	LPCi	7	
	AGp	Angular gyrus, posterior	39 (posterior)	LPCi	7	
	COp	Central opercular cortex, posterior	43	COp	43*	
	PCN	Precuneal cortex	7 (medial) (31)	MPC	7, 19 (18)	
	PO	Parietal opercular cortex	40	PO	7^†^	
	POGLi	Postcentral gyrus, lateral, inferior	3, 1, 2	POGL	3, 1, 2 (5)	
	POGLm	Postcentral gyrus, lateral, middle	3, 1, 2 (5)	POGL	3, 1, 2 (5)	
	POGLs	Postcentral gyrus, lateral, superior	3, 1, 2 (5)	POGL	3, 1, 2 (5)	
	POGm	Postcentral gyrus, medial	3, 1, 2 (5)	POGm	3, 1, 2 (5)	
	SGa	Supramarginal gyrus, anterior	40	LPCi	7	
	SGp	Supramarginal gyrus, posterior	40, 22	LPCi	7	
	SPLa	Superior parietal lobule, anterior	7 (5)	LPCs	7, 5	
	SPLp	Superior parietal lobule, posterior	7	LPCs	7, 5	
Temporal Lobe	H1	Heschl’s gyrus	41	STPp	22	
	INSa	Insular cortex, anterior	J ant (agranular)	INS	14, 15, 16 (agranular)^‡^	
	INSp	Insular cortex, posterior	J post (granular)	INS	13 (granular)^‡^	
	PP	Planum polare	22 (anterior)	STPa	22	
	PT	Planum temporale	22 (posterior), 42	STPp	22	
	T1a	Superior temporal gyrus, anterior	22 (anterior)^‡‡^	STG	22	
	T1p	Superior temporal gyrus, posterior	22 (posterior)	STG	22	
	T2a	Middle Temporal gyrus, anterior	21 (anterior)	ITG	20, 21 (19)	
	T2p	Middle temporal gyrus, posterior	21 (posterior), (22)	ITG	20, 21 (19)	
	T3a	Inferior temporal gyrus, anterior	20 (anterior)	ITG	20, 21 (19)	
	T3p	Inferior temporal gyrus, posterior	20 (posterior) (37)	ITG	20, 21 (19)	
	TFa	Temporal fusiform cortex, anterior	20, 36	ITG	20, 21 (19)	
	TFp	Temporal fusiform cortex, posterior	20, 36	ITG	20, 21 (19)	
	TO2	Middle temporal gyrus, temporo-occipital	37	ITG	20, 21 (19)	
	TO3	Inferior temporal gyrus, temporo-occipital	37	ITG	20, 21 (19)	
	TOF	Temporal occipital fusiform cortex	37	VMO	19, 20 (18)	
	TP	Temporal pole	38	TP	21, 22, 28	
Paralimbic Lobe	CGa_a	Cingulate gyrus, anterior, anterior part	33, 24	CGa	24 (23, 32)	
	CGa_p	Cingulate gyrus, anterior, posterior part	33, 24	CGa	24 (23, 32)	
	CGp	Cingulate gyrus, posterior	23, 31, 26, 29, 30	CGp	23, 26 (18, 19)	
	PAC	Paracingulate gyrus	32	F1dm	6, 9, 32	
	PHa	Parahippocampal gyrus, anterior	28, 34	PH	19, 20, 21, 27	
	PHp	Parahippocampal gyrus, posterior	27, 35	PH	19, 20, 21, 27	
	SC	Subcallosal cortex	25, 32 (24)	SC	24 (32)	

The major cytoarchitectonic designation of each parcellation unit is listed. Minor cytoarchitectonic regions within the parcellation units are enclosed in parentheses. Walker’s modification of Brodmann areas for the macaque frontal lobe are also listed for each parcellation unit in the frontal lobe.

*Brodmann (p. 127; Brodmann, 1909; Garey, 2006) notes that there is a structurally and morphologically comparable area 43 based on myeloarchitectonic and cytoarchitectonic criteria (p.127).

**This region appears to correspond to areas 6 and 43, but such correspondence is not definitive due to a lack of precise information in Brodmann (1909) and (Garey, 2006).

^†^This region is not explicitly specified in Brodmann (1909) and Garey (2006) and cytoarchitectonic correspondences were inferred based on textual and figural references.

^††^Note that Brodmann area 13 (insular) is not equivalent to Walker area 13 (orbitofrontal).

^‡^Includes dysgranular insular regions.

^‡‡^The borders established by Rademacher et al. (1993) were used for this ROI, however it should be noted that areas 38, 41, and 42 may also be involved.

## Discussion

In this study, we achieved three principal goals, as follows: (1) we developed a comparative methodology, referred to as HOA2.0-ComPaRe (Comparative Parcellation Reasoning), to parcellate brain structures in monkey and human brains with reference to an established cortical mapping framework (Brodmann, 1909; Garey, 2006); (2) we used this methodology in conjunction with accrued neuroscientific knowledge to delineate a more fine-grained set of parcellation units in both the human and the monkey HOA2.0 revisions; and (3) we produced a representative template brain atlas for each species (Supplementary material). It should be emphasized that the theoretical framework and methodology regarding neuroanatomy and atlasing are within the context of the CMA system of brain parcellation and morphometry that gave rise to the original HOA system in humans and monkeys (Frazier et al., 2005; Desikan et al., 2006; Makris et al., 2006a; Goldstein et al., 2007; Jenkinson et al., 2012; Rushmore et al., 2020a).

MRI-based volumetric analysis in humans began first at the CMA in the late 1980s with the systematic approach of Rademacher et al. (1992). This system of quantitative neuroanatomical analysis was advanced in subsequent studies (Caviness et al., 1996; Makris et al., 1999) and referred to as brain volumetrics (Caviness et al., 1999). Subsequently, this framework of volumetric analysis was used as the basis for the validation of the fully automated FreeSurfer approach (Fischl et al., 2002, 2004). It also gave rise to the HOA in humans and was the basis of the Desikan-Killiany atlas (Desikan et al., 2006; Jenkinson et al., 2012). The original brain volumetrics approach (Rademacher et al., 1992; Caviness et al., 1996, 1999) was manual and semiautomated. Although precise neuroanatomically, this approach was time and labor intensive, which limited its general applicability. The automation provided by FreeSurfer allowed more efficient morphometric brain processing, which then made possible the integration of brain morphometry with multimodal imaging (Fischl et al., 2002, 2004). Furthermore, FreeSurfer morphometric analysis became an integral component of multimodal imaging methodology as implemented in the current Human Connectome Project (HCP; Van Essen et al., 2012; Glasser et al., 2013). Recently, investigators such as Van Essen and Glasser (2018), Van Essen et al. (2019), and Rushmore et al. (2020a,b) have emphasized the importance of a comparative morphometric approach. Such an approach entails finer-grained, ontologically based parcellations related to established common cytoarchitectonic criteria (Brodmann, 1909; Garey, 2006). This allows for neuroanatomical comparability between cortical areas in non-human primates and humans (e.g., Brodmann, 1909; von Bonin and Bailey, 1947; Bailey and von Bonin, 1951; Pandya and Yeterian, 1996; Petrides and Pandya, 1999; Garey, 2006; Petrides et al., 2012). Moreover, this approach provides a basis for comparing structural connectivity between species (Rushmore et al., 2020a). Finally, such a comparative framework could be used to validate human structural connectivity on the basis of neuroanatomical tract tracing experiments in non-human primate models, as has been discussed in recent publications (Van Essen and Glasser, 2018; Van Essen et al., 2019; Rushmore et al., 2020a,b).

### Structural considerations

In this study, we developed a more fine-grained, comparative cortical parcellation for the human and the rhesus monkey brain. This was carried out in the frontal, parietal, and occipital lobe in both species, in the temporal lobe in the monkey, and in the insula in the human. The rationale for this approach was established with the original human HOA. Essentially, it is based on consistent and reliable morphological features that are visible in MRI and approximate underlying structural anatomy such as sulci and gyri, as well as fiber tracts and nuclei (Brodmann, 1909; Rademacher et al., 1992; Filipek et al., 1994; Caviness et al., 1996, 1999; Makris et al., 1999; Garey, 2006). The revised parcellation units as defined here constitute more discrete nodes and thus can allow for more precise delineation of distinct structural neural networks. This comparative neuroanatomical framework allows the formulation of testable hypotheses for both the human and the macaque that can provide insight on local features such as specific fiber tracts as well as the hierarchical organization of the central nervous system (e.g., Pandya and Yeterian, 1985; Rademacher et al., 1992; Mesulam, 2000).

### Ontology, sizes and scales

The nervous system, and specifically the study of brain connectivity, has been conceived to have three main levels of organization. The macroscale level of connectional analysis refers to the connections of one brain region to another brain region. At this level, brain regions are seen essentially as black boxes comprising multiple populations of neurons, each with potentially different patterns of connections (Swanson and Lichtman, 2016). The next finer level of organization, the mesoscale, specifies the connections between distinct groups of neurons within the regional level. These groups can be defined on the basis of neuronal class (e.g., pyramidal neurons, interneurons) or on the basis of a subregional organizational scheme (e.g., columnar, minicolumnar, laminar-specific). Finally, the microscale level refers to connections between individual neurons within subregions (e.g., Elston, 2007; Defelipe, 2011).

These three levels of brain organization relate to connectivity, but may also be applied to the analysis of brain structure *per se* (Amunts and Zilles, 2015). At the macroscopic level, brain regions are defined by specific criteria. These regions could represent parcellation units or regions of interest, as delineated in the present study, or be defined according to other criteria e.g., Brodmann cytoarchitectonic areas (Brodmann, 1909; Garey, 2006) or Vogt myeloarchitectonic areas (Nieuwenhuys, 2013; Nieuwenhuys and Broere, 2020). At the more detailed level of the mesoscale, brain regions may be divided into populations of neurons, defined as such through subregional or specific cellular classifications. The microscopic level involves the delineation of individual neurons and their associated morphologies (e.g., Defelipe, 2011). From this tripartite perspective, the parcellation units defined in the present study, as well as in other cerebral cortical formulations (e.g., Tzourio-Mazoyer et al., 2002; Fischl et al., 2004; Desikan et al., 2006; Fan et al., 2016; Glasser et al., 2016), are consistent with a macroscale level. The subdivision of existing parcellation units, for example, that of the F2 parcellation unit of Caviness et al. (1996) into F2a, F2m, and F2p, does not represent a change in the level of analysis from macroscale to mesoscale, but rather a refinement at a macroscale level of organization.

When structural comparisons of ROIs across species are made, a key factor is that they be performed at the same scale of analysis to ensure ontological comparability. Other critical factors for ontological comparability include the histological composition of these ROIs, their functions, as well as their structural and functional connectivity. The cytoarchitectonic schema of Brodmann provides a foundation for the common comparative and ontological criteria underlying comparisons across scales. For example, the precentral gyrus and the posterior cingulate gyrus in the Brodmann formulation are comparable in human and macaque not only at a macroscale level, but also at the microscale level. More precisely, the existence of Betz cells in the precentral gyrus defines Brodmann area 4 in both human and macaque, and the cytoarchitectonic characteristics of Brodmann area 23 in the posterior cingulate gyrus are consistent in humans and non-human primates (Brodmann, 1909; Garey, 2006). Furthermore, the cytoarchitectonic features of the frontal lobe can be compared in humans and macaques (Pandya and Yeterian, 1985, 1996; Petrides et al., 2012; Yeterian et al., 2012). Such structural correspondences can be extended to the entire cerebral cortex (e.g., Brodmann, 1909; von Bonin and Bailey, 1947; Bailey and von Bonin, 1951; Garey, 2006). The cytoarchitectonic areas of Brodmann can be viewed as macroscale conglomerates of individual cells essentially equivalent to a PU or ROI in a neuroimaging context. Given the comparability at the macroscale and microscale levels in Brodmann’s comparative approach, we expect this approach to constitute a solid foundation for MRI-based comparative parcellation schemas such as the one described herein. Moreover, structural pathways within the brain arise from and are organized in relation to cytoarchitectonic areas, which underlies the comparability of structural connectivity across species. As neuroimaging technology advances to allow the acquisition of datasets at ultra-high spatial resolution, we foresee that cytoarchitectonic characterization of ROIs will be achieved using MRI (e.g., Roland et al., 1997; Calabrese et al., 2015; Amunts et al., 2020). Thus, the HOA-ComPaRe approach can become a powerful means for structural and functional brain analysis in basic and clinical neuroscience.

### Functional considerations

The present revised HOA parcellation system is relevant for understanding the functional architecture of the cerebral cortex in both humans and monkeys, with respect to functional localization as well as functional connectivity. The subdivision of frontal and parietal cortical areas has been shown to be necessary to disambiguate distinct functional networks. For instance, the anterior portion of the middle frontal gyrus has distinct functional and network properties involved with attentional processes when compared to more posterior portions, which are more closely tied to working memory (Cieslik et al., 2013). Although the division of regions within gyri has been emphasized in reports of structural neuroanatomy (e.g., Morosan et al., 2005; Caspers et al., 2013, 2014; Lorenz et al., 2017), task-related functional activation cannot be localized precisely in specific cytoarchitectonic areas using neuroimaging methods. This is because technological and analytical techniques have not yet achieved the level of resolution to precisely identify cytoarchitectonic areas in the *in vivo* human brain, with the exception of the primary visual cortex (BA17) (e.g., Rademacher et al., 1993; Hinds et al., 2009). Nevertheless, the HOA2.0-ComPaRe approach could facilitate studies of functional architectonic organization across species, the importance of which has been emphasized by several investigators (e.g., Fox et al., 2005; Vincent et al., 2007; Yeo et al., 2011; Buckner and Krienen, 2013; Krienen et al., 2014).

### Clinical considerations

The original human HOA system has been utilized in applied clinical research to better understand the neural basis of many major neurological and psychiatric disorders (e.g., Makris et al., 2006a, 2008a,b; Seidman et al., 2006; Frazier et al., 2007; Wrase et al., 2008; Blood et al., 2010). The most common neurological conditions examined using this system include disorders characterized by visible and quantifiable lesions, such as strokes or brain tumors (e.g., Caviness et al., 2002). Other neurological conditions involve neurodegenerative disorders such as Huntington’s disease and neurodevelopmental syndromes such as autism (e.g., Herbert et al., 2003; Rosas et al., 2003; Hong et al., 2018). In psychiatry, the human HOA system has been used to analyze an array of conditions including substance use disorders (e.g., alcohol use disorder), schizophrenia, bipolar disorder, major depressive disorder and ADHD (e.g., Makris et al., 2006a,b, 2008a,b, 2009; Seidman et al., 2006; Frazier et al., 2007; Wrase et al., 2008; Blood et al., 2010). Furthermore, this system has been employed to localize neuromodulation approaches for the treatment of neurological and psychiatric disorders such as Huntington’s disease and obsessive compulsive disorder (OCD) (Rosas et al., 2001, 2003; Cannistraro et al., 2007; Makris et al., 2016; Hong et al., 2018). A more precise parcellation system thus provides a more anatomically accurate and efficient means by which brain structure in individuals with pathological conditions can be assessed and monitored during treatment. Furthermore, the comparative approach is critical to brain circuit mapping using novel treatments in psychiatry such as transcranial magnetic stimulation (Ning et al., 2022; Salerno et al., 2022). Overall, the scope of disorders that can be studied with this system of analysis demonstrates its feasibility, broad applicability, and versatility.

### Limitations and future studies

The nature of our parcellation system is topographical and quantitative and based on anatomical landmarks of the individual brain. Furthermore, it is comparative between human and non-human primate brains. A key limitation of this and any other parcellation schema using MRI-based anatomical landmark identification is that the parcellation units do not necessary correspond precisely to neuroanatomically defined areas, e.g., to a given Brodmann area or areas (e.g., Sanides, 1969; Rademacher et al., 1993). Although there is a consistent relationship between morphology (i.e., sulci) and histology for primary cortical areas (Rademacher et al., 1993), this relationship is much less clear in unimodal and especially heteromodal association regions (e.g., Sanides, 1969; Rademacher et al., 1993). In other words, the correspondences between parcellation units in the neuroimaging domain and the cytoarchitectonic maps of Brodmann are necessarily approximate due to the inability of MRI technology to visualize structure at a histological level *in vivo* (e.g., Elston and Garey, 2009, 2013; Amunts and Zilles, 2015; Glasser et al., 2016). Despite this limitation, relating morphology to cytoarchitectonic areas has been established practice in MRI-based morphometry since its inception (e.g., Rademacher et al., 1992, 1993; Caviness et al., 1996; Fischl et al., 2004). More importantly, such correspondences have demonstrated value in MRI-based basic and clinical research (e.g., Makris et al., 2006a; Seidman et al., 2006; Frazier et al., 2007; Makris et al., 2008a,b; Wrase et al., 2008; Blood et al., 2010). Nevertheless, caution should be applied when generating correspondences between parcellation units and cytoarchitectonic domains and using them to interpret experimental or clinical results. It is expected that the future availability of MRI technology and protocols with greater spatial resolution will improve the accuracy of parcellation, and may ultimately depict cytoarchitectonic features more precisely. This, in turn, will lead to the development of more anatomically driven parcellation schemas that can be applied in generating anatomically curated datasets to be used in deep learning for the generation of more structurally accurate atlases. Moreover, we expect this approach to be useful in future studies using MRI morphometry to address issues such as inter-individual variability, sex differences, hemispheric dominance, and aging in cerebral cortical structure in basic and clinical research. The present approach, which incorporates manual tracing that preserves anatomical features such as sulci and gyri in individual brains, is particularly appropriate for studying inter-individual anatomical differences.

## Conclusion

We present a comparative system to relate human and monkey brain structure, grounded in a framework that we term HOA2.0-Comparative Parcellation Reasoning (HOA2.0-ComPaRe). This system provides revisions for the human HOA (hHOA2.0) and the monkey HOA (mHOA2.0) comparative methods for the parcellation of individual brains and the generation of HOA2.0 brain atlases. HOA2.0-ComPaRe was developed to address the need in the field of anatomical neuroimaging for an explicitly comparative morphometric methodology in brain parcellation within a common histologically referenced and MRI-based methodological framework for human and monkey brains. This framework was refined and expanded in light of accrued information on brain structure and function. We also provided a representative atlas for each species based on a single subject. We expect the hHOA2.0 and the mHOA2.0 to be used in neuroimaging for the purposes of improved localization and cortical parcellation in studies of structural and functional connectivity. Finally, we expect these revised and refined cortical parcellations to serve as the basis for training deep learning algorithms to produce finer-grained MRI-based atlases of the cerebral cortex in both humans and macaques.

## Data availability statement

The original contributions presented in this study are included in the article/Supplementary material, further inquiries can be directed to the corresponding author/s.

## Ethics statement

The work involving the human participant was reviewed and approved by the Massachusetts General Hospital IRB. The participant provided their informed consent to participate in this study. The animal work was reviewed and approved by the IACUCs at Boston University and Massachusetts General Hospital.

## Author contributions

NM, EY, and RR wrote the first draft of the manuscript. All authors read, revised, and approved the final manuscript.
