# Supplementary figures and images for: HOA2.0-ComPaRe: A next generation Harvard-Oxford Atlas comparative parcellation reasoning method for human and macaque individual brain parcellation and atlases of the cerebral cortex
Source: Front Neuroanat. 2022 Nov 10;16:1035420. doi: 10.3389/fnana.2022.1035420 (PMC9684647; doi:10.3389/fnana.2022.1035420)

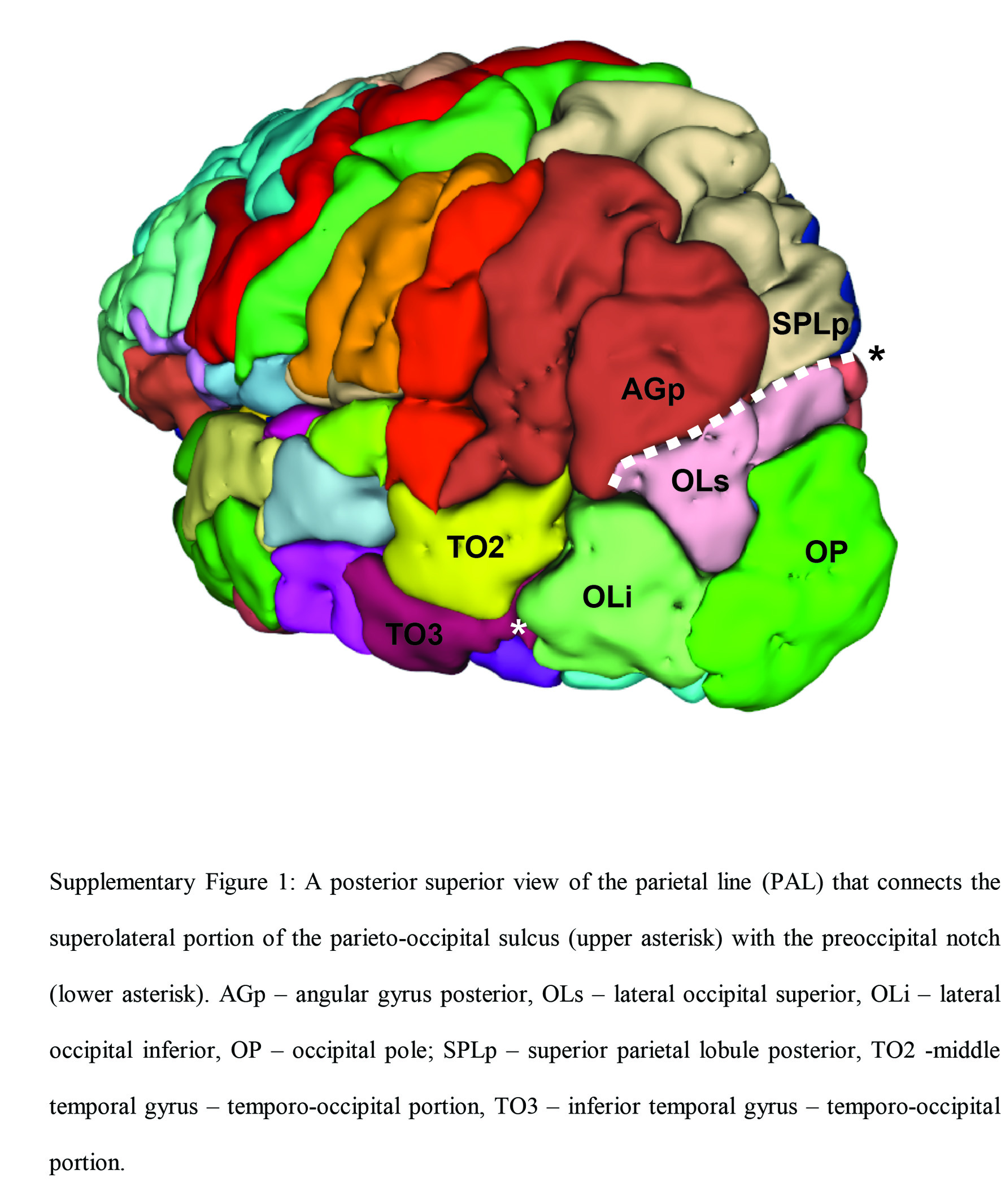

Supplement: Supplementary file 1 [file Image_1.JPEG]

# The Macaque Harvard-Oxford Atlas (mHOA) Single Subject Atlas

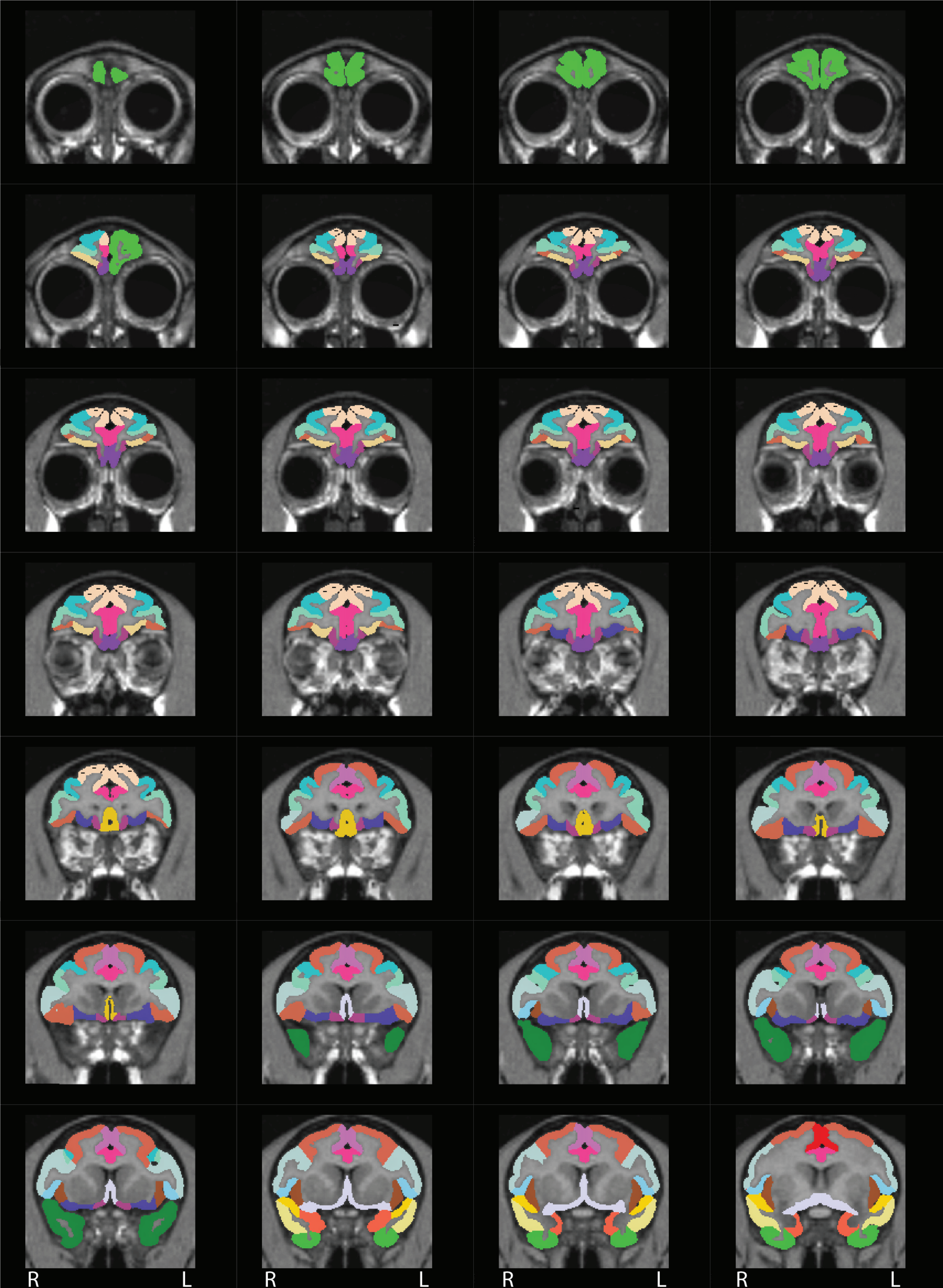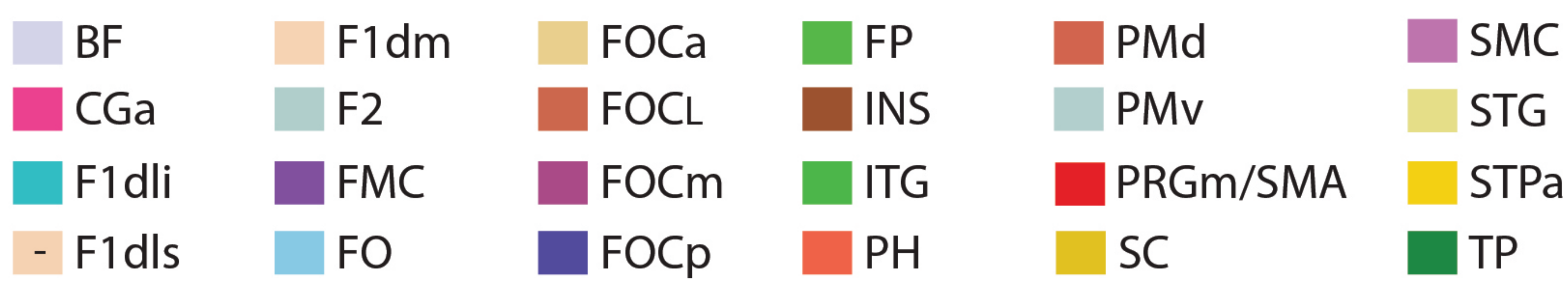

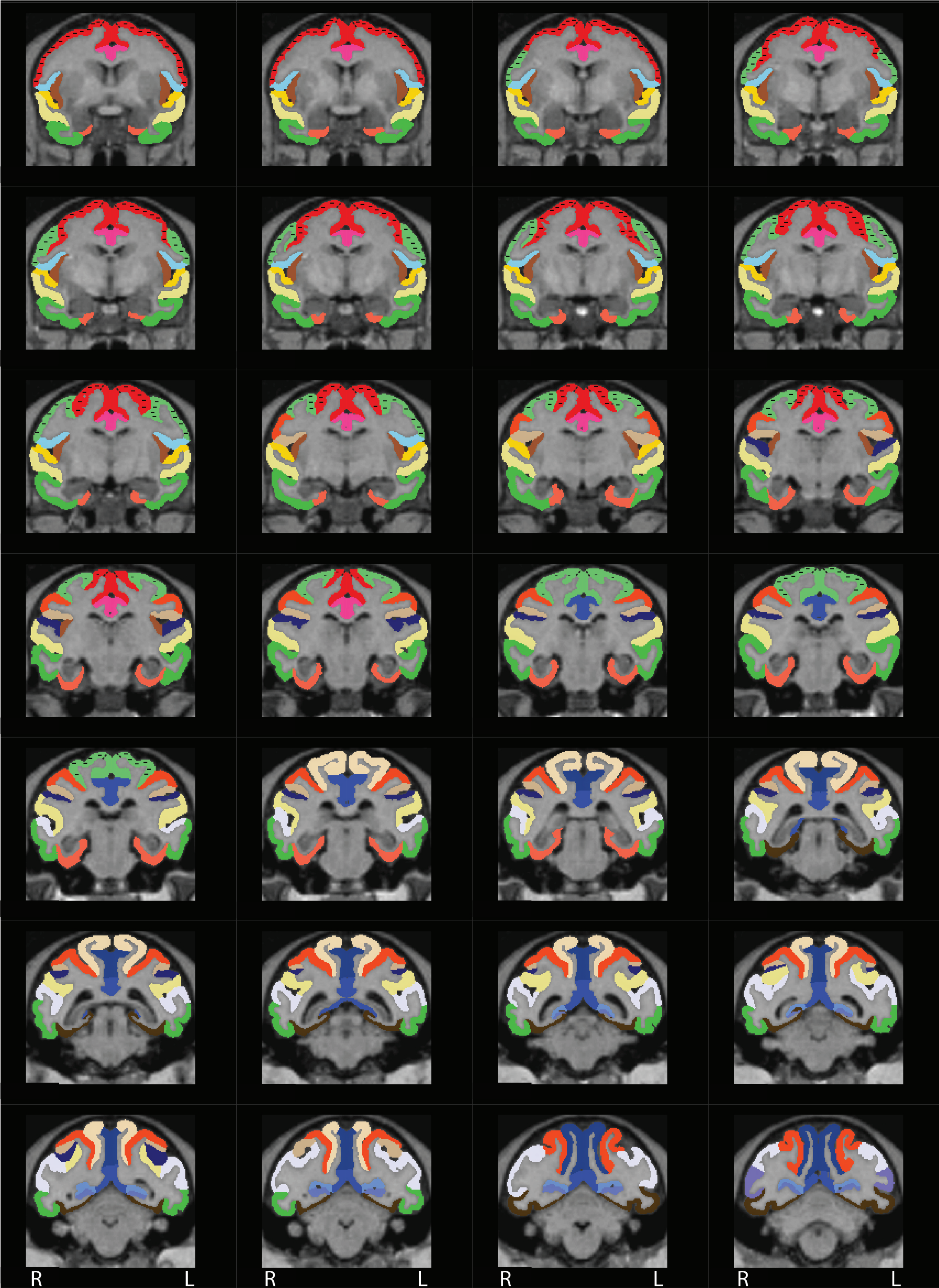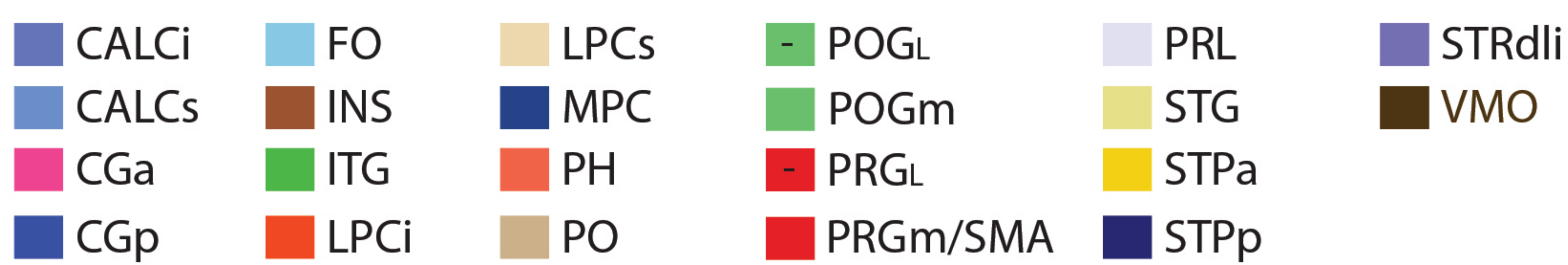

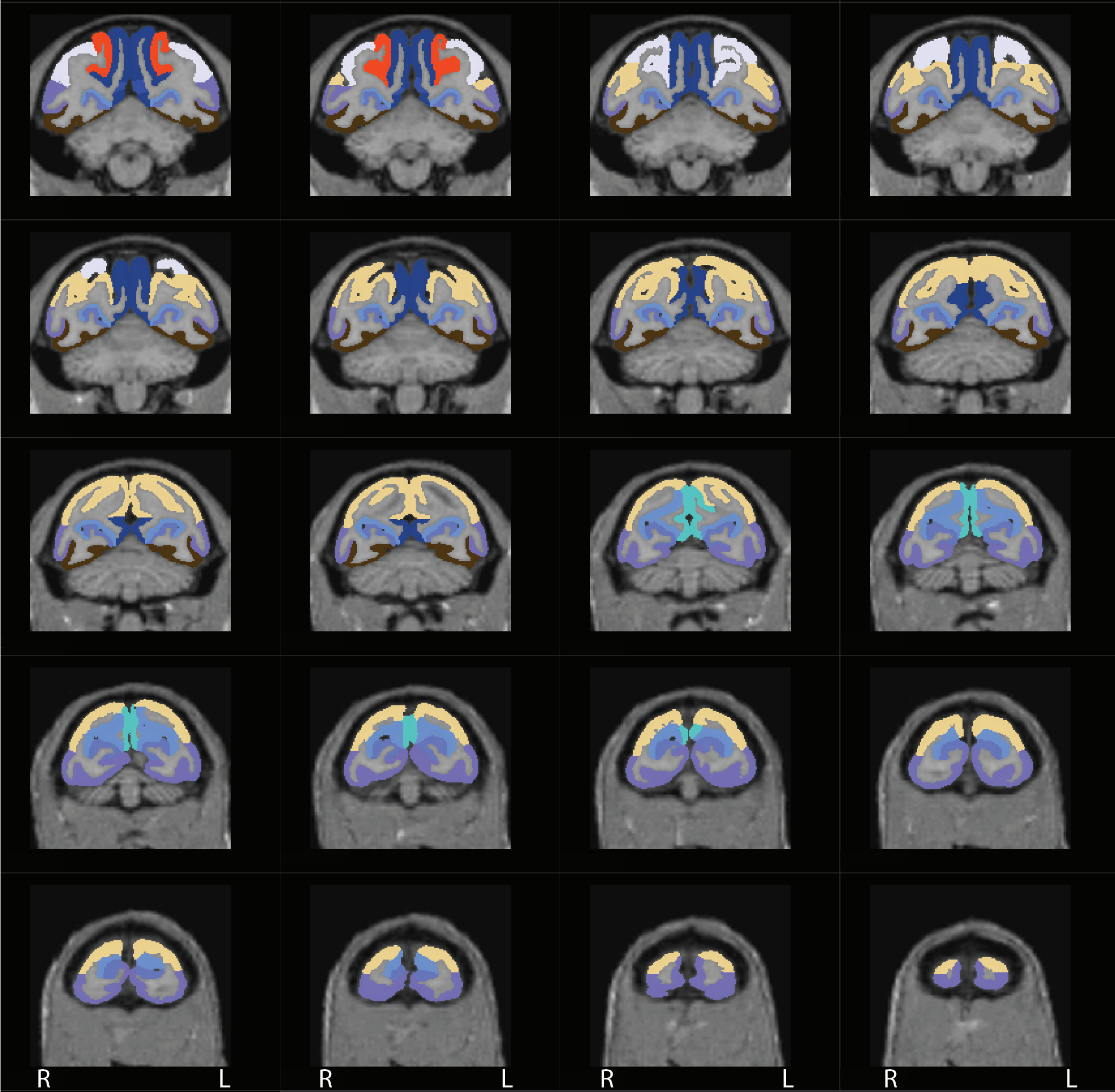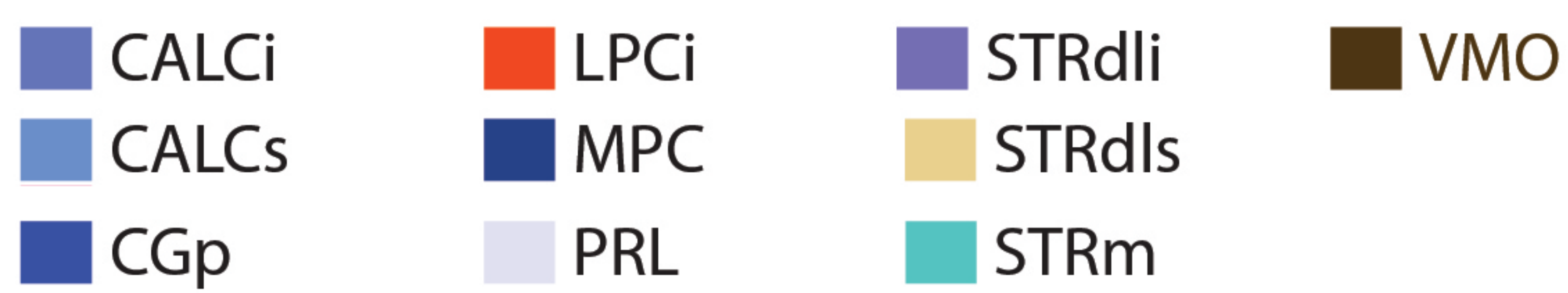

Supplement: Supplementary file 4 [file Data_Sheet_1.PDF]

# The Human Harvard-Oxford Atlas (hHOA) Single Subject Atlas

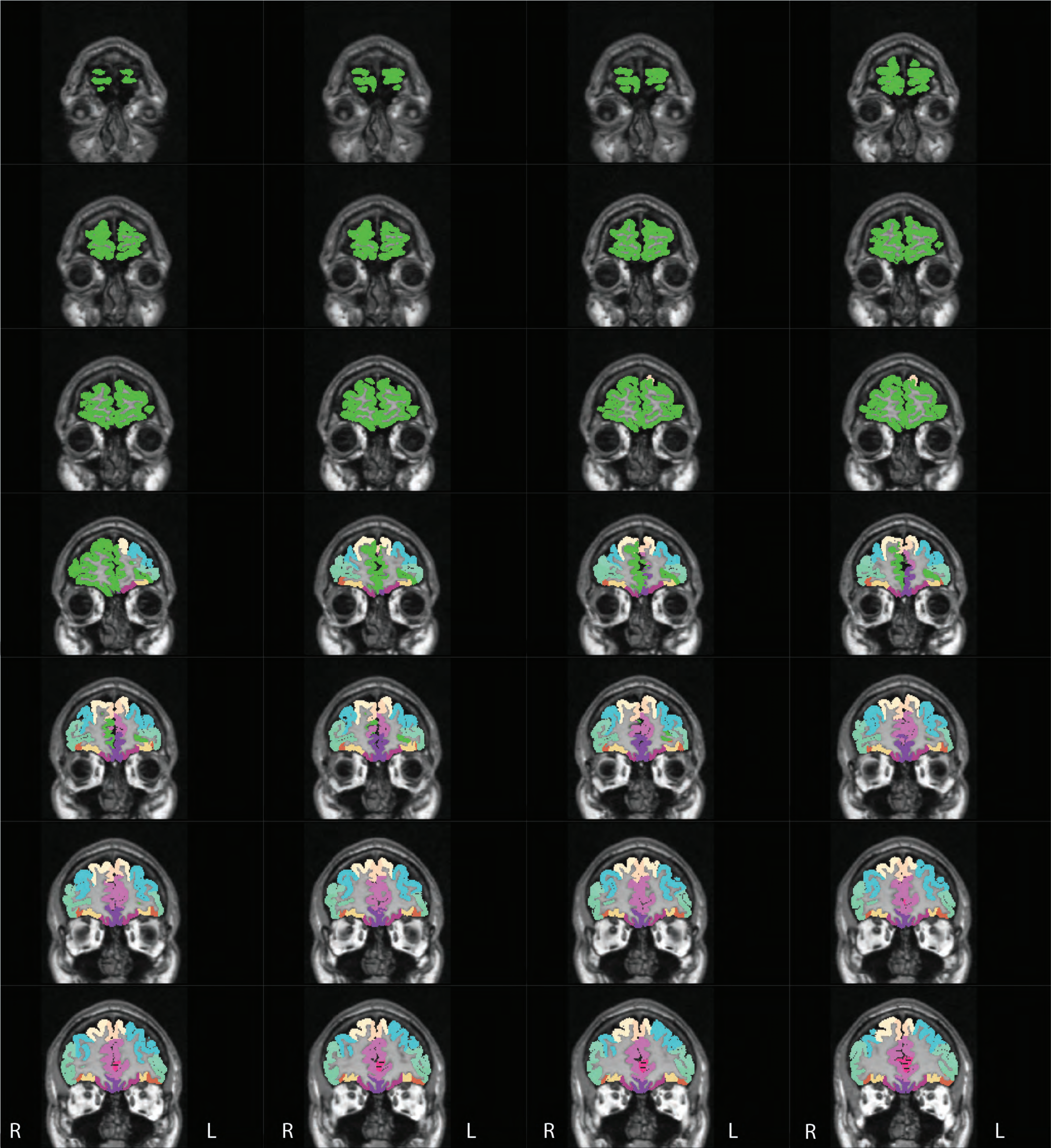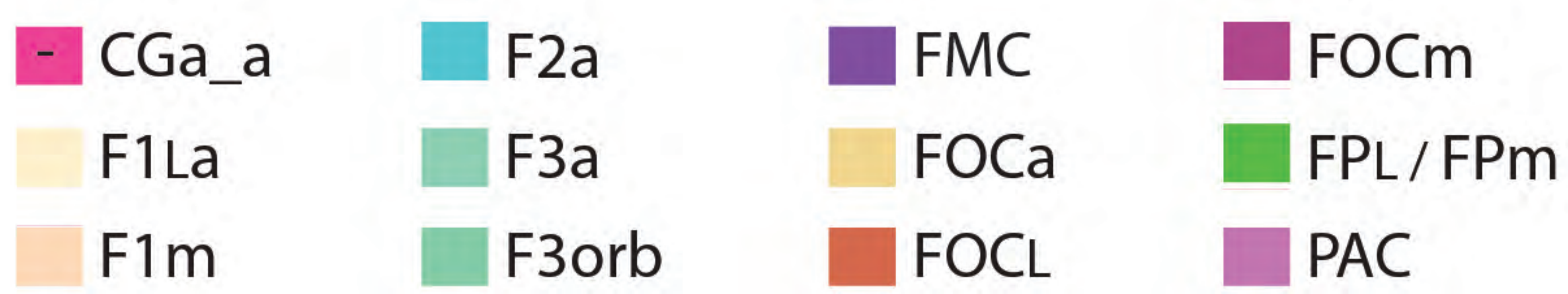

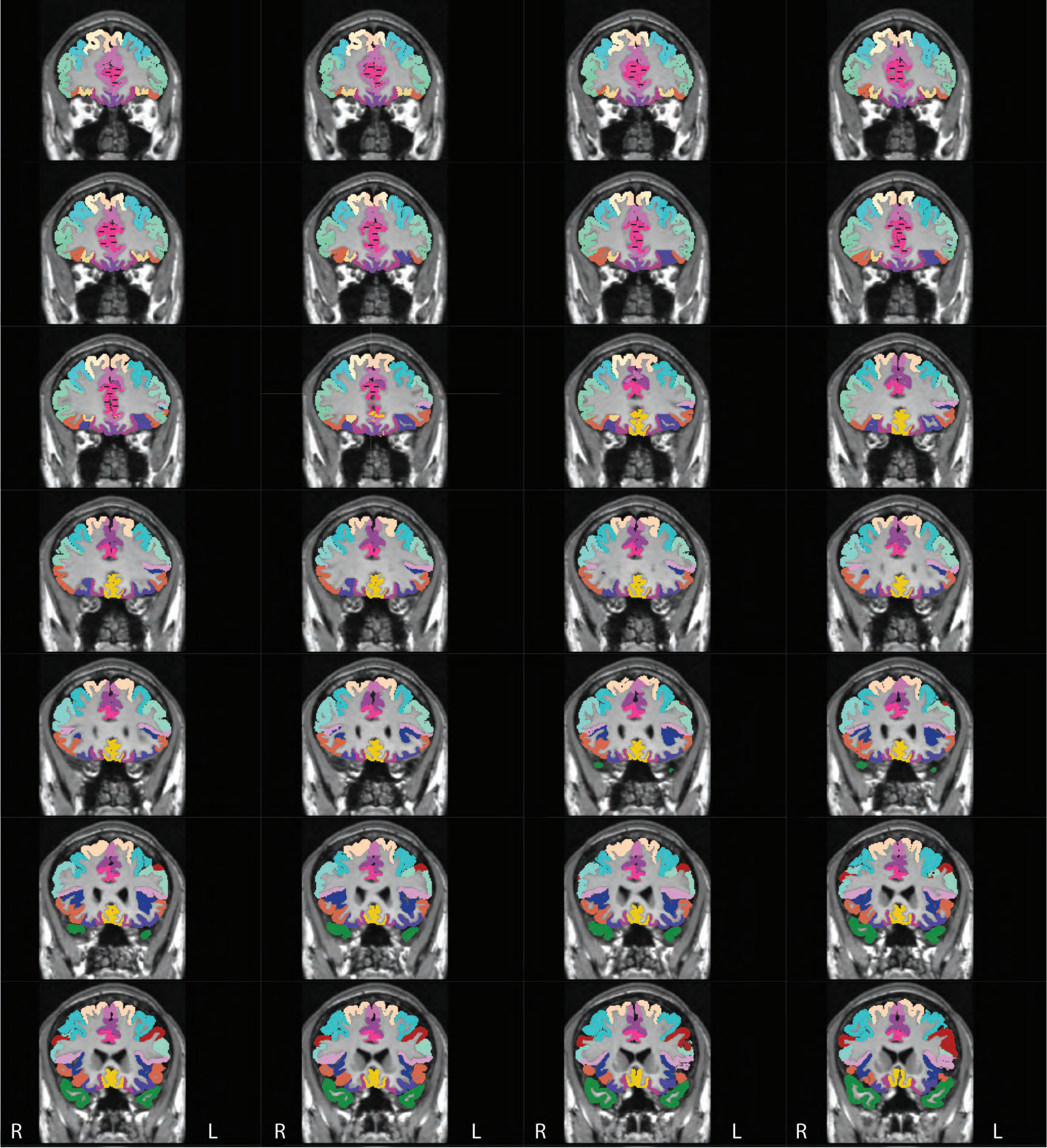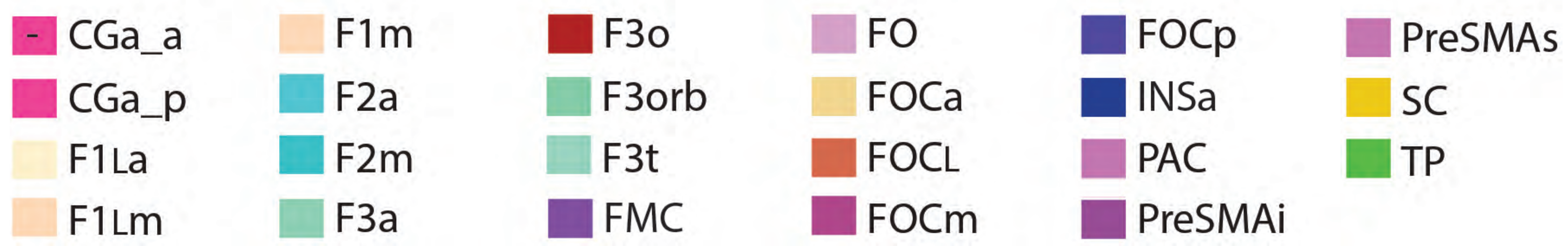

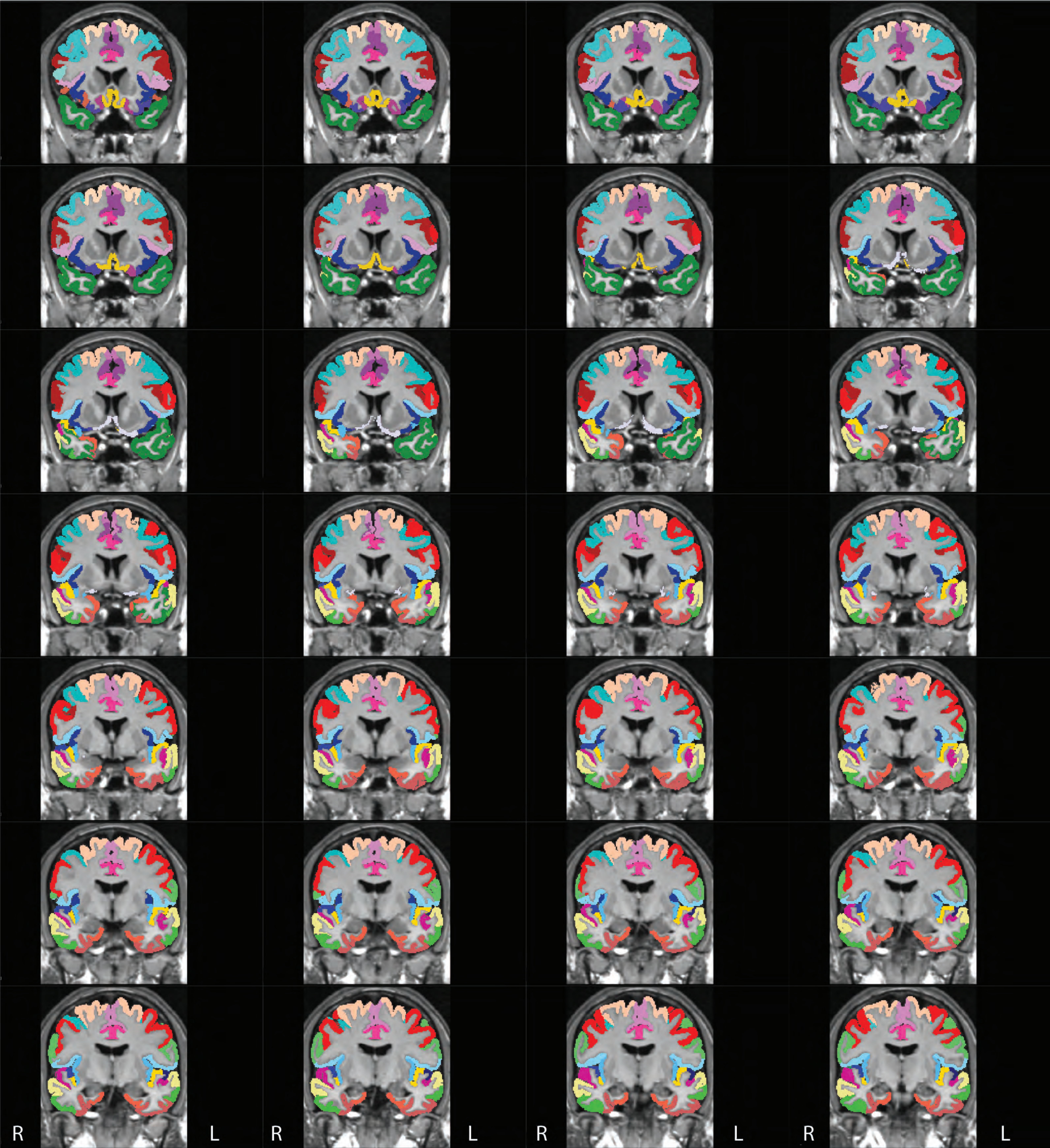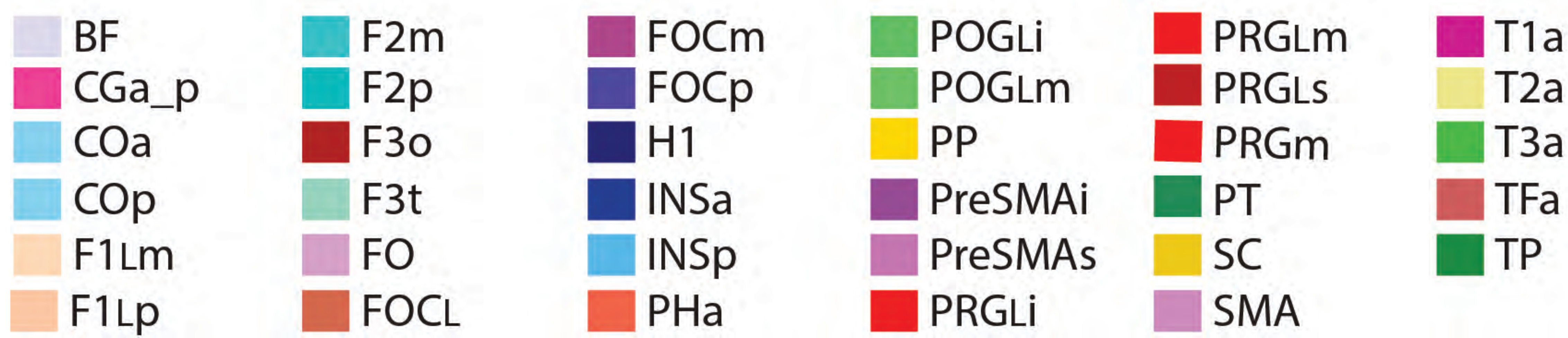

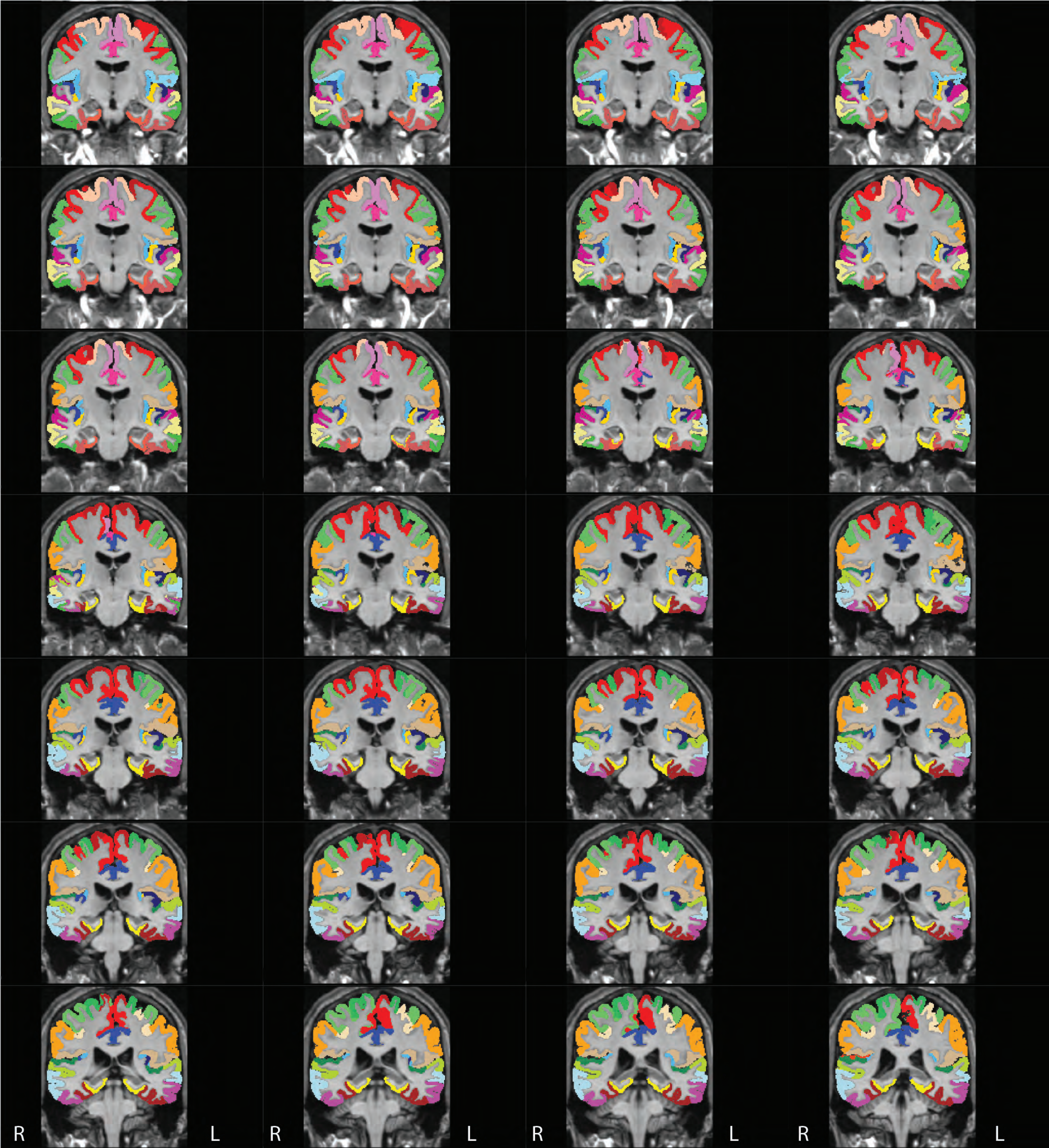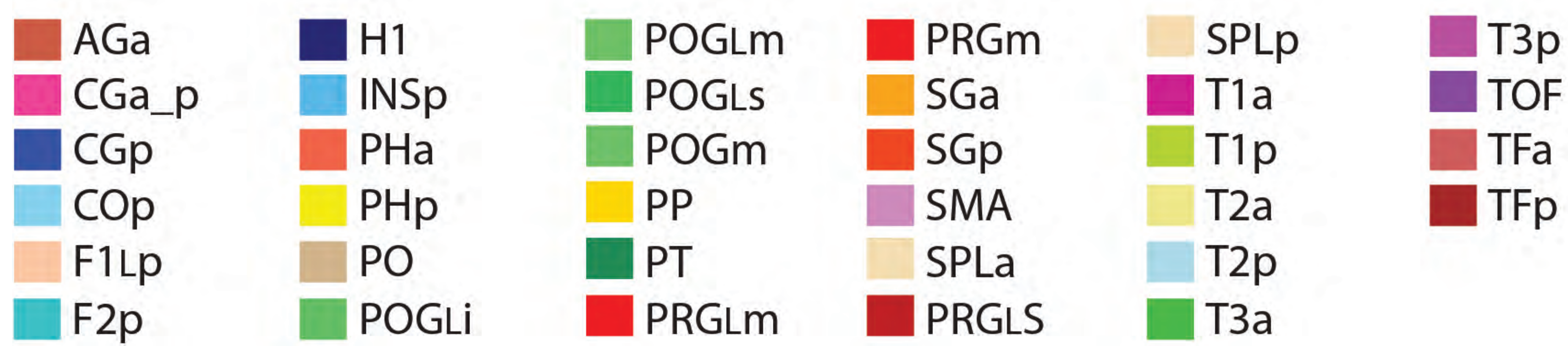

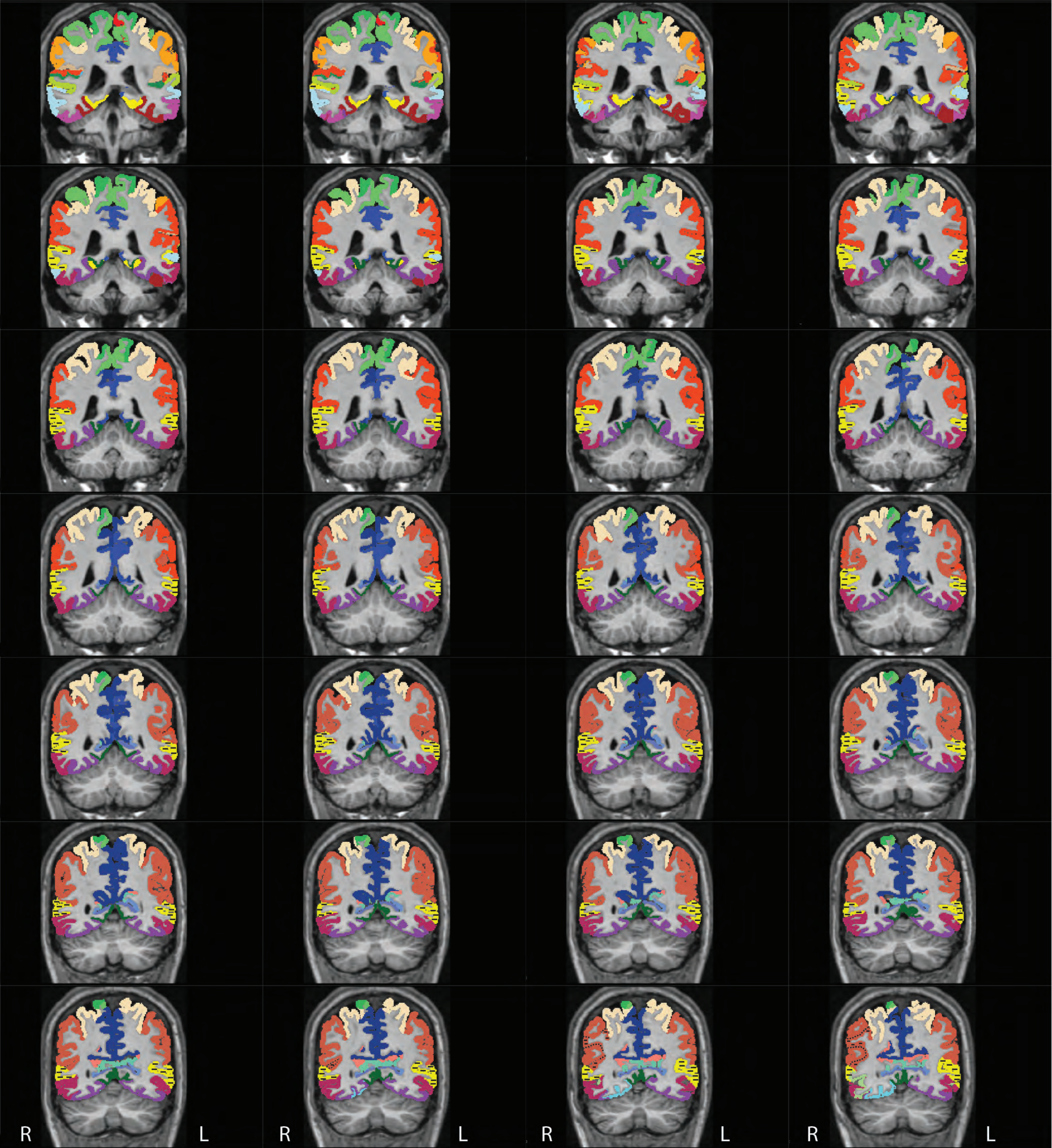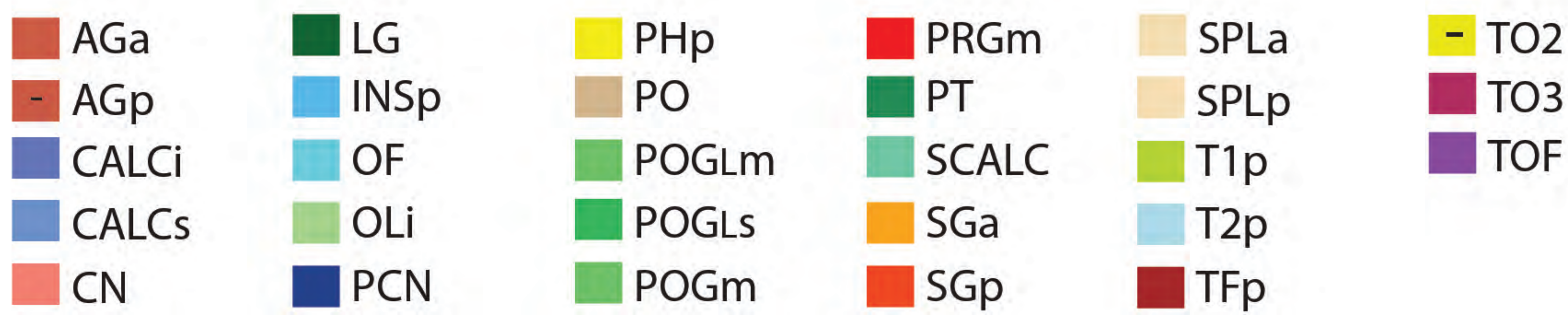

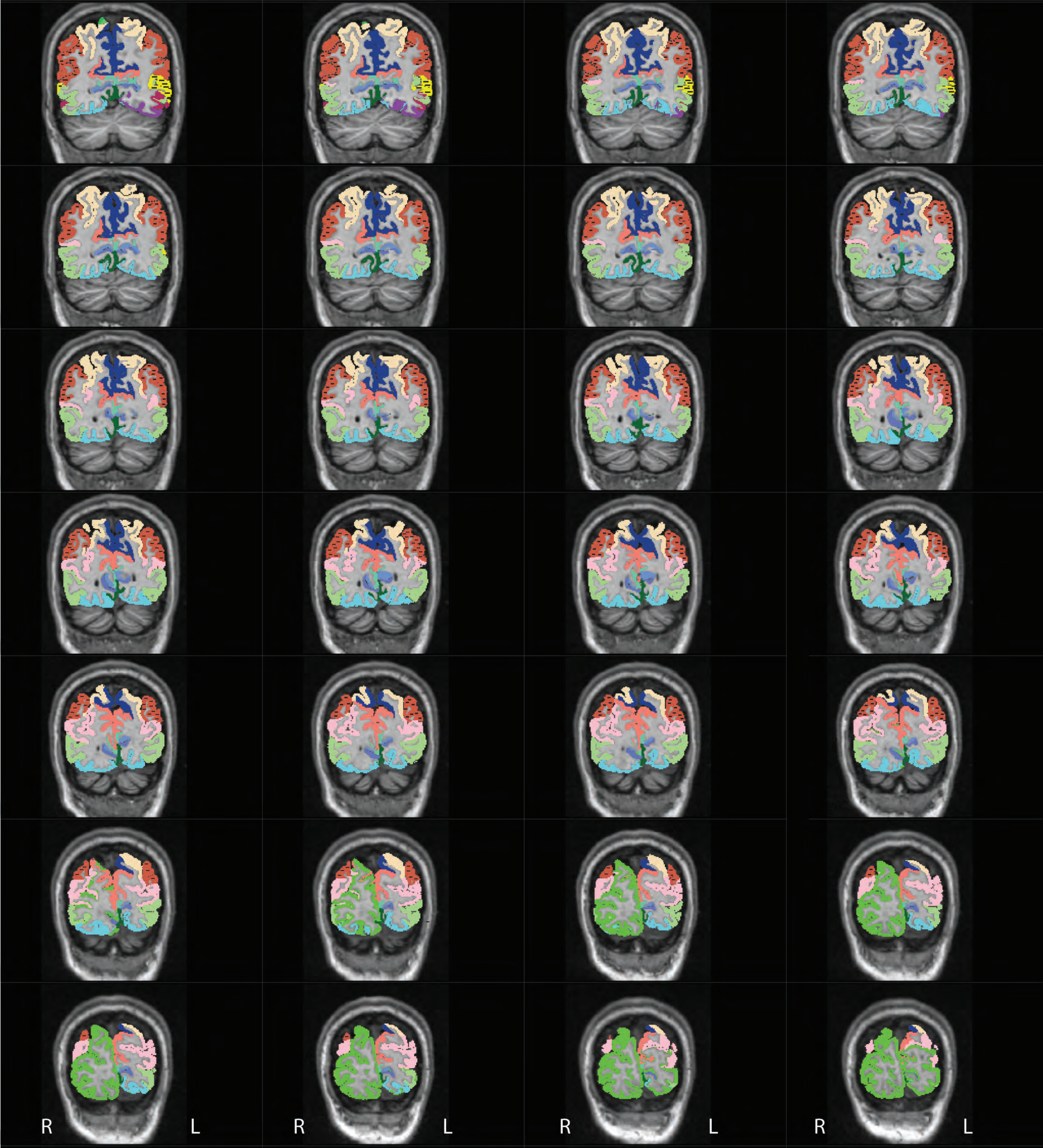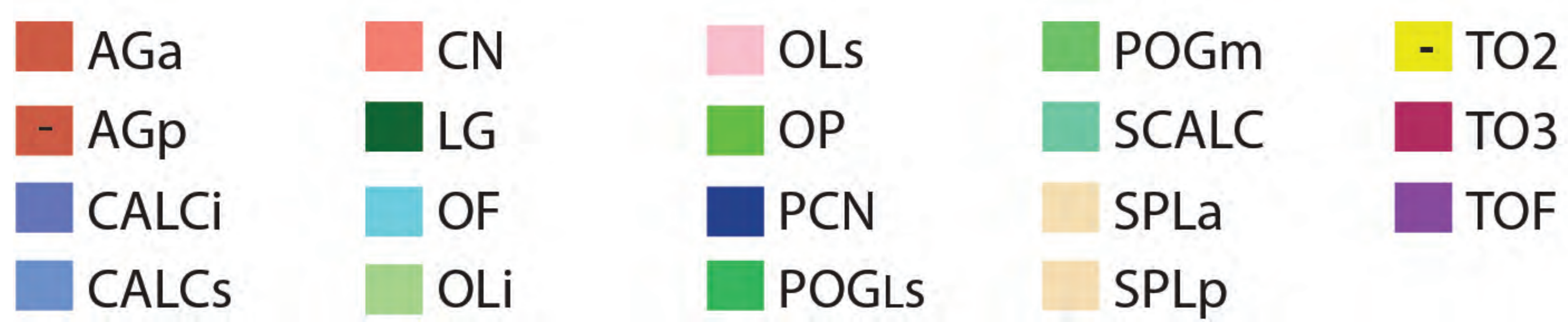

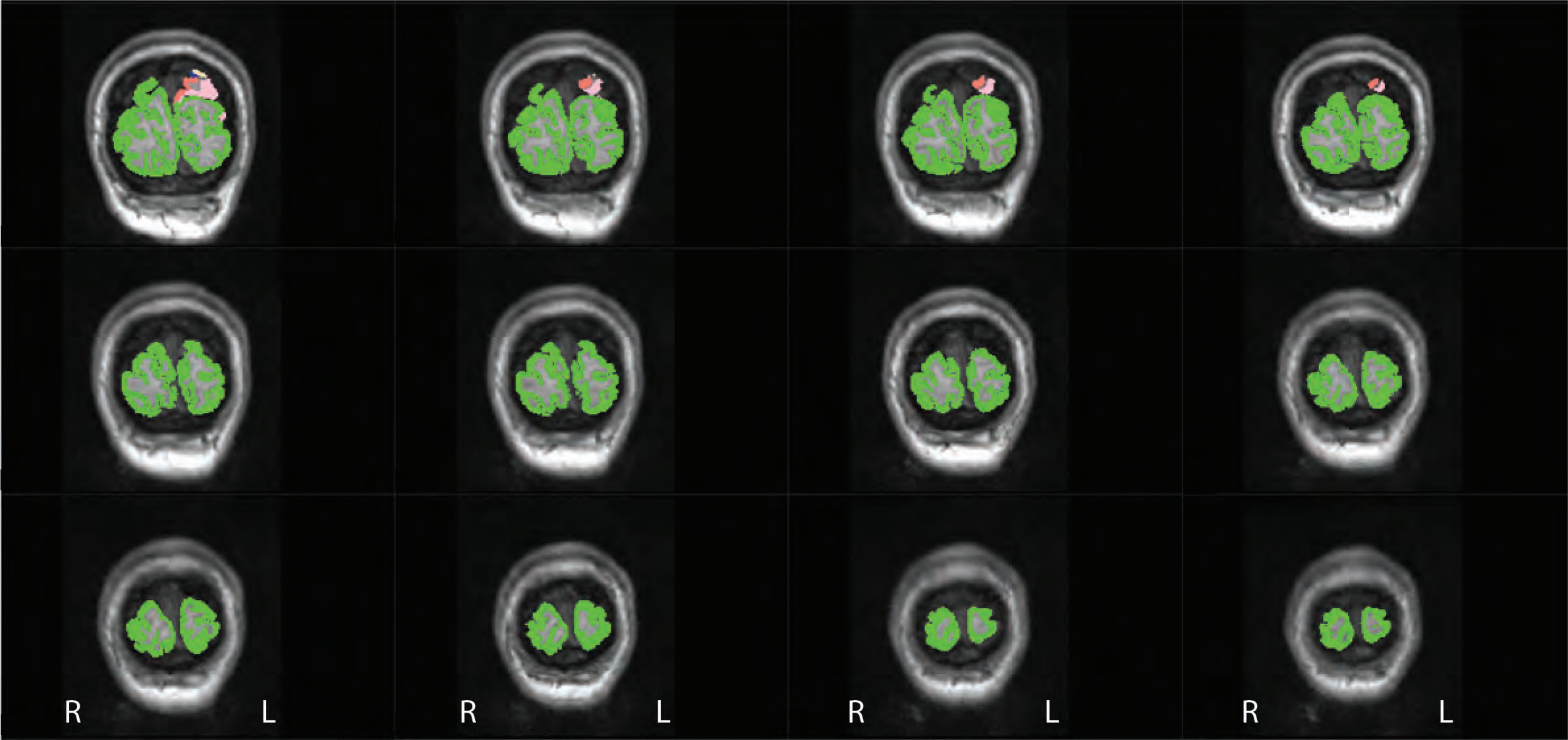

Supplement: Supplementary file 5 [file Data_Sheet_2.PDF]
