# Supplementary material for: HOA2.0-ComPaRe: A next generation Harvard-Oxford Atlas comparative parcellation reasoning method for human and macaque individual brain parcellation and atlases of the cerebral cortex
Source: Front Neuroanat. 2022 Nov 10;16:1035420. doi: 10.3389/fnana.2022.1035420 (PMC9684647; doi:10.3389/fnana.2022.1035420)
Supplement: Supplementary file 2 [file Table_1.DOCX]

Supplementary Table 1: Functional Correspondences of human Harvard Oxford (hHOA) Atlas Parcellation Units

| **Lobe** | **PU** | **hHOA Parcellation Unit** | **Functional Region** |
| --- | --- | --- | --- |
| Frontal Lobe | COa | Central Opercular Cortex, anterior | HSA |
|  | F1La | Superior Frontal Gyrus, lateral, anterior | HFA |
|  | F1Lm | Superior Frontal Gyrus, lateral, middle | MA (PMC), HFA |
|  | F1Lp | Superior Frontal Gyrus, lateral, posterior | MA (PMC) |
|  | F1m | Superior Frontal Gyrus, medial | HFA |
|  | F2a | Middle Frontal Gyrus, anterior | HFA |
|  | F2m | Middle Frontal Gyrus, middle | MA (FEF, PMC), HFA |
|  | F2p | Middle Frontal Gyrus, posterior | MA (FEF, PMC) |
|  | F3a | Inferior Frontal Gyrus, anterior | HFA |
|  | F3o | Inferior Frontal Gyrus, pars opercularis | MA |
|  | F3orb | Inferior Frontal Gyrus, pars orbitalis | HFA |
|  | F3t | Inferior Frontal Gyrus, pars triangularis | HFA |
|  | FMC | Frontal Medial Cortex | HFA |
|  | FO | Frontal Opercular Cortex | HFA, MA |
|  | FOCa | Frontal Orbital Cortex, anterior | HFA |
|  | FOC_L_ | Frontal Orbital Cortex, lateral | HFA |
|  | FOCm | Frontal Orbital Cortex, medial | HFA |
|  | FOCp | Frontal Orbital Cortex, posterior | HFA |
|  | FP_L_ | Frontal Pole, lateral | HFA |
|  | FPm | Frontal Pole, medial | HFA |
|  | PreSMAi | Pre-Supplementary Motor Area, inferior | MA (PreSMA) |
|  | PreSMAs | Pre-Supplementary Motor Area, superior | MA (PreSMA) |
|  | PRGLi | Precentral Gyrus, lateral, inferior | M1 |
|  | PRGLm | Precentral Gyrus, lateral, middle | M1 |
|  | PRGLs | Precentral Gyrus, lateral, superior | M1 |
|  | PRGm | Precentral Gyrus, medial | M1 |
|  | SMA | Supplementary Motor Area | MA (SMA) |
|  |  |  |  |
| Occipital Lobe | CALCi | Intracalcarine Cortex, inferior | V1 |
|  | CALCs | Intracalcarine Cortex, superior | V1 |
|  | CN | Cuneal Cortex | VA (V2, V3, VA) |
|  | LG | Lingual Gyrus | VA (V2, V3) |
|  | OF | Occipital Fusiform Gyrus | VA (V4) |
|  | OLi | Lateral Occipital Cortex, inferior | VA (V5) |
|  | OLs | Lateral Occipital Cortex, superior | VA |
|  | OP | Occipital Pole | V1, VA (V2) |
|  | SCALC | Supracalcarine Cortex | V1, VA (V2) |
|  |  |  |  |
| Parietal Lobe | AGa | Angular Gyrus, anterior | HPA |
|  | AGp | Angular Gyrus, posterior | HPA |
|  | COp | Central Opercular Cortex, posterior | HSA |
|  | PCN | Precuneal Cortex | SA, SSA, HPA |
|  | PO | Parietal Opercular Cortex | SA, AA, HPA |
|  | POGLi | Postcentral Gyrus, lateral, inferior | S1, SA |
|  | POGLm | Postcentral Gyrus, lateral, middle | S1, SA |
|  | POGLs | Postcentral Gyrus, lateral, superior | S1, SA |
|  | POGm | Postcentral Gyrus, medial | S1, SA |
|  | SGa | Supramarginal Gyrus, anterior | HPA |
|  | SGp | Supramarginal Gyrus, posterior | HPA |
|  | SPLa | Superior Parietal Lobule, anterior | SA, HPA |
|  | SPLp | Superior Parietal Lobule, posterior | HPA |
|  |  |  |  |
| Temporal Lobe | H1 | Heschl's Gyrus | A1 |
|  | INSa | Insular Cortex, anterior | POA |
|  | INSp | Insular Cortex, posterior | POA |
|  | PP | Planum Polare | POA |
|  | PT | Planum Temporale | AA1 |
|  | T1a | Superior Temporal Gyrus, anterior | AA2, AA3 |
|  | T1p | Superior Temporal Gyrus, posterior | AA1 |
|  | T2a | Middle Temporal Gyrus, anterior | VA |
|  | T2p | Middle Temporal Gyrus, posterior | VA |
|  | T3a | Inferior Temporal Gyrus, anterior | VA |
|  | T3p | Inferior Temporal Gyrus, posterior | VA |
|  | TFa | Temporal Frontal Cortex, anterior | PHA, VA |
|  | TFp | Temporal Frontal Cortex, posterior | PHA, VA |
|  | TO2 | Middle Temporal Gyrus, temporo-occipital | VA |
|  | TO3 | Inferior Temporal Gyrus, temporo-occipital | VA |
|  | TOF | Temporal Occipital Fusiform Cortex | PHA, VA |
|  | TP | Temporal Pole | POA |
|  |  |  |  |
| Paralimbic Lobe | CGa_a | Cingulate Gyrus, anterior, anterior part | PHA |
|  | CGa_p | Cingulate Gyrus, anterior, posterior part | PHA |
|  | CGp | Cingulate Gyrus, posterior | PHA |
|  | PAC | Paracingulate Gyrus | PHA |
|  | PHa | Parahippocampal Gyrus, anterior | PHA |
|  | PHp | Parahippocampal Gyrus, posterior | PHA |
|  | SC | Subcallosal Cortex | PHA |

**Abbreviations:**

A1 – Primary auditory cortex

V1 – Primary visual cortex

S1 – Primary somatosensory cortex

M1 – Primary motor cortex

AA – Auditory association cortex (indicated as 1, 2, 3)

VA – Visual association cortex (indicated as 1, 2, 3, 4 ,5)

SA – Somatosensory association area

SSA – Supplementary somatosensory area

MA – Motor association cortex (specified as PMC, premotor cortex; SMA, supplementary motor area;

FEF, frontal eye field)

HFA – Heteromodal frontal association cortex

HSA – Heteromodal subcentral association cortex

HPA – Heteromodal parietal association cortex

POA – Paralimbic olfactocentric association cortex

PHA – Paralimbic hippocampocentric association cortex
