# Supplementary material for: HOA2.0-ComPaRe: A next generation Harvard-Oxford Atlas comparative parcellation reasoning method for human and macaque individual brain parcellation and atlases of the cerebral cortex
Source: Front Neuroanat. 2022 Nov 10;16:1035420. doi: 10.3389/fnana.2022.1035420 (PMC9684647; doi:10.3389/fnana.2022.1035420)
Supplement: Supplementary file 3 [file Table_2.DOCX]

Supplementary Table 2: Functional Correspondences of macaque Harvard-Oxford Atlas (mHOA) Parcellation Units

| **Lobe** | **PU** | **mHOA Parcellation Unit** | **Functional Region** |
| --- | --- | --- | --- |
| Frontal Lobe | COa | Central Opercular Cortex, anterior | HAS |
|  | F1dli | Middle Frontal Gyrus | HFA |
|  | F1dls | Superior Frontal Gyrus | HFA |
|  | F1dm | Superior Frontal Gyrus, medial | HFA |
|  | F2 | Inferior Frontal Gyrus | HFA |
|  | FMC | Frontal Medial Cortex | HFA |
|  | FOCa | Frontal Orbital Cortex, anterior | HFA |
|  | FOCL | Frontal Orbital Cortex, lateral | HFA |
|  | FOCm | Frontal Orbital Cortex, medial | HFA |
|  | FOCp | Frontal Orbital Cortex, posterior | HFA |
|  | FP | Frontal Pole | HFA |
|  | PMd | Premotor Cortex, dorsal | MA |
|  | PMv | Premotor Cortex, ventral | MA |
|  | PreSMA | Pre-Supplementary Motor Area | MA |
|  | PRGL | Precentral Gyrus, lateral | M1 |
|  | PRGm/SMA | Precentral Gyrus, medial / Supplementary Motor Area | M1 |
|  |  |  |  |
| Occipital Lobe | CALCi | Intracalcarine Cortex, inferior | V1 |
|  | CALCs | Intracalcarine Cortex, superior | V1 |
|  | PRL | Prelunate Gyrus | VA |
|  | STRdli | Striate Cortex, dorsolateral, inferior | V1 |
|  | STRdls | Striate Cortex, dorsolateral, superior | V1 |
|  | STRm | Striate Cortex, medial | V1 |
|  | VMO | Ventromedial Occipital Cortex | VA |
|  |  |  |  |
| Parietal Lobe | COp | Central Opercular Cortex, posterior | S1, SA |
|  | LPCi | Lateral Parietal Cortex, inferior | SA, HPA |
|  | LPCs | Lateral Parietal Cortex, superior | SA, HPA |
|  | MPC | Medial Parietal Cortex | SA |
|  | PO | Parietal Opercular Cortex | SA, HPA |
|  | POGL | Postcentral Gyrus, lateral | S1 |
|  | POGm | Postcentral Gyrus, medial | S1 |
|  |  |  |  |
| Temporal Lobe | INS | Insular Cortex | POA |
|  | ITG | Inferior Temporal Gyrus | VA |
|  | STG | Superior Temporal Gyrus | VA, AA |
|  | STPa | Supratemporal Plane, anterior | A1, AA |
|  | STPp | Supratemporal Plane, posterior | A1, AA |
|  | TP | Temporal Pole | POA |
|  |  |  |  |
| Paralimbic Lobe | CGa | Cingulate Gyrus, anterior | PHA |
|  | CGp | Cingulate Gyrus, posterior | PHA |
|  | PH | Parahippocampal Gyrus | PHA |
|  | SC | Subcallosal Cortex | PHA |

**Abbreviations:**

A1 – Primary auditory cortex

V1 – Primary visual cortex

S1 – Primary somatosensory cortex

M1 – Primary motor cortex

AA – Auditory association cortex (indicated as 1, 2, 3)

VA – Visual association cortex (indicated as 1, 2, 3, 4, 5)

SA – Somatosensory association area

SSA – Supplementary somatosensory area

MA – Motor association cortex (specified as PMC, premotor cortex; SMA, supplementary motor area;

FEF, frontal eye field)

HFA – Heteromodal frontal association cortex

HSA – Heteromodal subcentral association cortex

HPA – Heteromodal parietal association cortex

POA – Paralimbic olfactocentric association cortex

PHA – Paralimbic hippocampocentric association cortex
